# Di‐Zinc–Aryl Complexes: CO_2_ Insertions and Applications in Polymerisation Catalysis

**DOI:** 10.1002/chem.201701013

**Published:** 2017-05-05

**Authors:** Charles Romain, Jennifer A. Garden, Gemma Trott, Antoine Buchard, Andrew J. P. White, Charlotte K. Williams

**Affiliations:** ^1^ Department of ChemistryChemistry Research LaboratoryUniversity of Oxford12 Mansfield RoadOxfordOX2 3TAUK; ^2^ Department of ChemistryImperial College LondonLondonSW7 2AZUK; ^3^ EastCHEM School of ChemistryUniversity of EdinburghEdinburghEH9 3FJUK; ^4^ Department of ChemistryUniversity of BathBathBA2 7AYUK

**Keywords:** CO_2_ insertion, organozinc catalysts, reactivity studies, ring-opening polymerisation, ring-opening copolymerisation, zinc

## Abstract

Two new di‐zinc–aryl complexes, [LZn_2_Ph_2_] and [LZn_2_(C_6_F_5_)_2_], coordinated by a diphenol tetraamine macrocyclic ligand have been prepared and fully characterised, including by single‐crystal X‐ray diffraction experiments. The complexes’ reactivities with monomers including carbon dioxide, cyclohexene oxide, phthalic anhydride, isopropanol and phenol were investigated using both experimental studies and density functional theory calculations. In particular, [LZn_2_Ph_2_] readily inserts carbon dioxide to form a carboxylate, at 1 bar pressure, whereas [LZn_2_(C_6_F_5_)_2_] does not react. Under these conditions [LZn_2_Ph_2_] shows moderate activity in the ring‐opening copolymerisation of cyclohexene oxide/carbon dioxide (TOF=20 h^−1^), cyclohexene oxide/phthalic anhydride (TOF=33 h^−1^) and the ring‐opening polymerisations of *rac*‐lactide (TOF=99 h^−1^) and *ϵ*‐caprolactone (TOF=5280 h^−1^).

## Introduction

Since their original discovery by Frankland in 1848,^1^ organometallic zinc compounds have become a well‐established component of the synthetic chemists’ toolbox. They have been successfully applied as stoichiometric reagents in Negishi cross‐coupling reactions,^2^ metal–halogen exchange,^3^ the alkylation of trifluoromethyl ketones^4^ and the epoxidation of enones.^5^ Zinc is an attractive choice of metal for catalysis,^6^ due to its low toxicity, low cost and lack of colour and redox chemistry. Homogeneous zinc catalysts show promise in reactions including the ring‐opening polymerisation (ROP) of cyclic esters,^7^ the formation of cyclic carbonates,^8^ aldol reactions^9^ and hydroamination reactions.^10^ They have been particularly effective as catalysts for CO_2_/epoxide ring‐opening copolymerisation (ROCOP), which provides a useful method of adding value to captured CO_2_.^11^ Some of the most active and selective catalysts are zinc complexes coordinated by β‐diiminate or phenoxy‐amine ligand scaffolds.^12^ With some of these different catalyst systems, short‐chain telechelic polycarbonates have been observed,^13^ which are potentially useful for chain extension reactions to form block copolymers,^14^ polyurethanes,^15^ or nanomaterials.^16^ The presence of such α,ω‐dihydroxyl end‐capped polymers is generally attributed to the presence of diols, formed through the reaction of epoxides with trace water, which act as chain‐transfer agents during polymerisation. Darensbourg and co‐workers recently gleaned further insight into the nature of this reaction, and established that this hydrolysis is catalysed by the polymerisation catalyst [(salen)Co(O_2_CCF_3_)], in both CO_2_/cyclohexene oxide (CHO) and CO_2_/propylene oxide (PO) ROCOP systems. Careful spectroscopic studies demonstrated that these [(salen)Co(O_2_CCF_3_)]‐catalysed hydrolysis reactions occur prior to any initiation of CO_2_/epoxide ROCOP, as the catalyst is initially occupied in the conversion of epoxides to diols.14a Fundamental reactivities of polymerisation catalysts towards oxygenated small‐molecules in ROCOP systems, including alcohols, carbon dioxide and other monomers, are of particular relevance to further understand the reactions occurring with chain‐transfer agents, and for the preparation of new catalysts for CO_2_/epoxide ROCOP, and so we studied the reactivity of zinc catalyst systems with a range of small molecules. Controlling the nature of the bond between the metal and the initiating group or growing polymer chain end is of key interest in polycarbonate synthesis,^17^ and has led to the development of “switchable” zinc catalysts, which can catalyse both the ROP of lactones and the ROCOP of epoxides with CO_2_ or anhydrides, thus enabling the controlled synthesis of block copolymers from a mixture of monomers.^18^

Considering the general reactivity of zinc–alkyl complexes, there are a number of reports of reactions with alcohols or carboxylic acids.^19^ The insertion of CO_2_ into Zn–alkoxide bonds has also been studied in depth.11b Some examples relevant to catalysis include the reversible insertion of CO_2_ into di‐zinc–alkoxide complexes based on a macrocyclic bis(anilido)tetraimine ligand, to form di‐zinc carbonate and mixed carbonate/alkoxide products.^20^ Considering BDI–Zn (BDI=β‐diiminate) complexes, which are well‐studied catalysts for CO_2_/epoxide ROCOP, zinc‐alkoxides rapidly insert CO_2_, whilst the epoxide coordination and ring‐opening is an equilibrium process.11b, 19c Despite these studies, the reaction of Zn–alkyl complexes with carbon dioxide remains much less explored,^21^ and the initial reactivity of such complexes in the presence of CO_2_, epoxide and diols is still not well understood. Kinetic studies have shown that CO_2_ insertion occurs rapidly for a series of zinc hydride complexes, to form the corresponding zinc formate complexes, in which the reaction kinetics were limited by the rate of CO_2_ dissolution in toluene solvent (*k*_obs_=0.033 m min^−1^).^22^

**Scheme 1:**
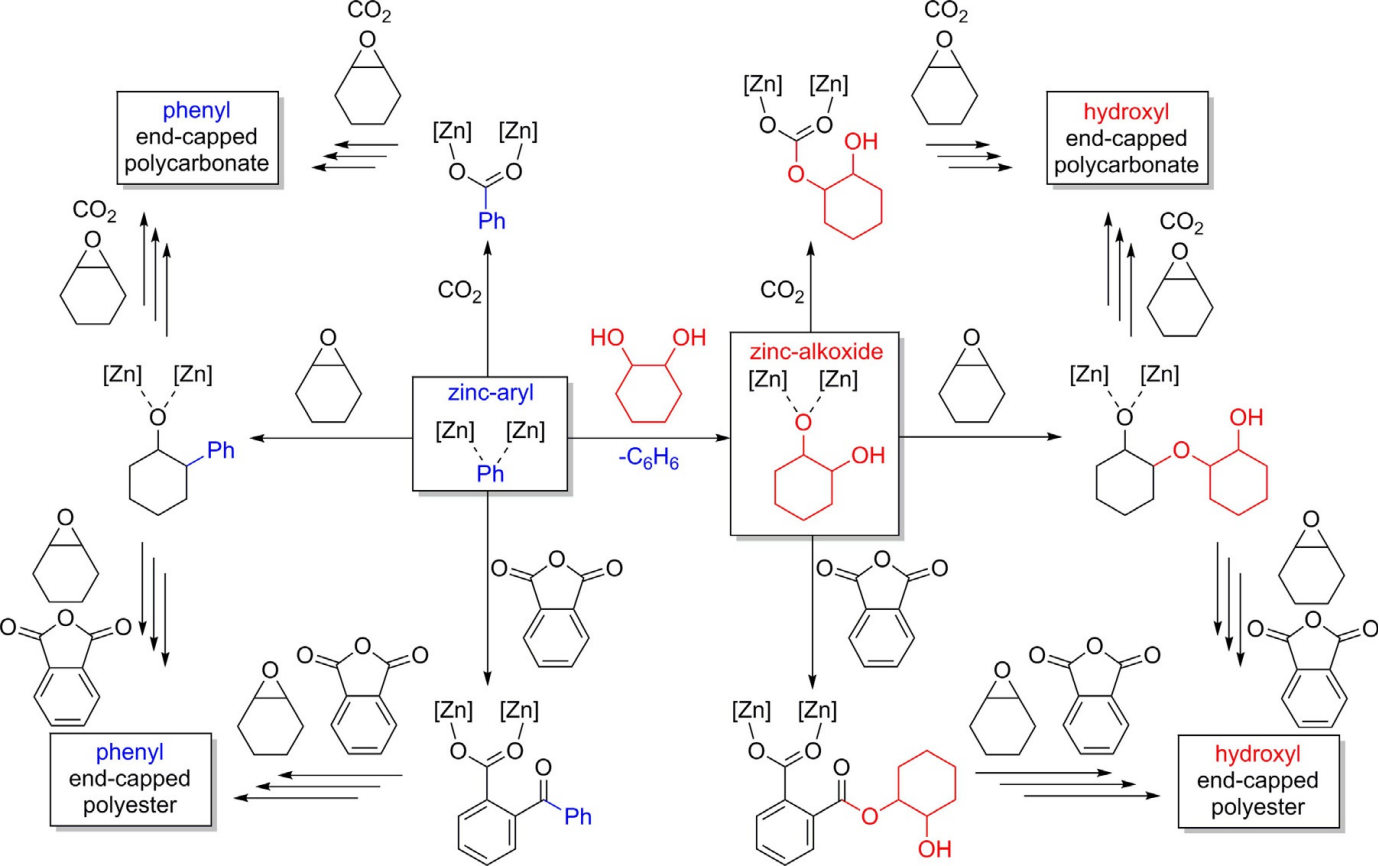
Reactivity overview of the plausible reactions of di‐zinc–aryl complexes with monomers CO_2_, CHO and PA, in the presence of chain‐transfer agent 1,2‐cyclohexenediol.

Recently, some of us reported a diphenol tetraamine‐based macrocyclic ligand that was used to prepare a series of dinuclear catalysts,13d, 23 including di‐zinc carboxylate compounds.12b, 24 These complexes showed activities for both the ROCOP of CO_2_/epoxide and of epoxide/anhydride, and were notable in being able to selectively polymerise at just 1 bar pressure of CO_2_.^25^ Here, we apply the same ligand and investigate the potential to prepare di‐zinc–bis(aryl) precatalysts. To gain insight into the reactions that may occur between such precatalysts and the key monomers or chain‐transfer agents present during polymerisation (Scheme 1), the reactivity of the complexes towards stoichiometric epoxide (CHO), phthalic anhydride (PA), CO_2_ and alcohols was explored. The effect of electron‐withdrawing substituents on the aryl co‐ligand was also compared, through experimental and computational comparisons, between di‐zinc–bis(phenyl) and di‐zinc–bis(pentafluorophenyl) complexes.

## Results and Discussion

### Complex synthesis (LZn_2_Ph_2_ and LZn_2_(C_6_F_5_)_2_)

**Scheme 2:**
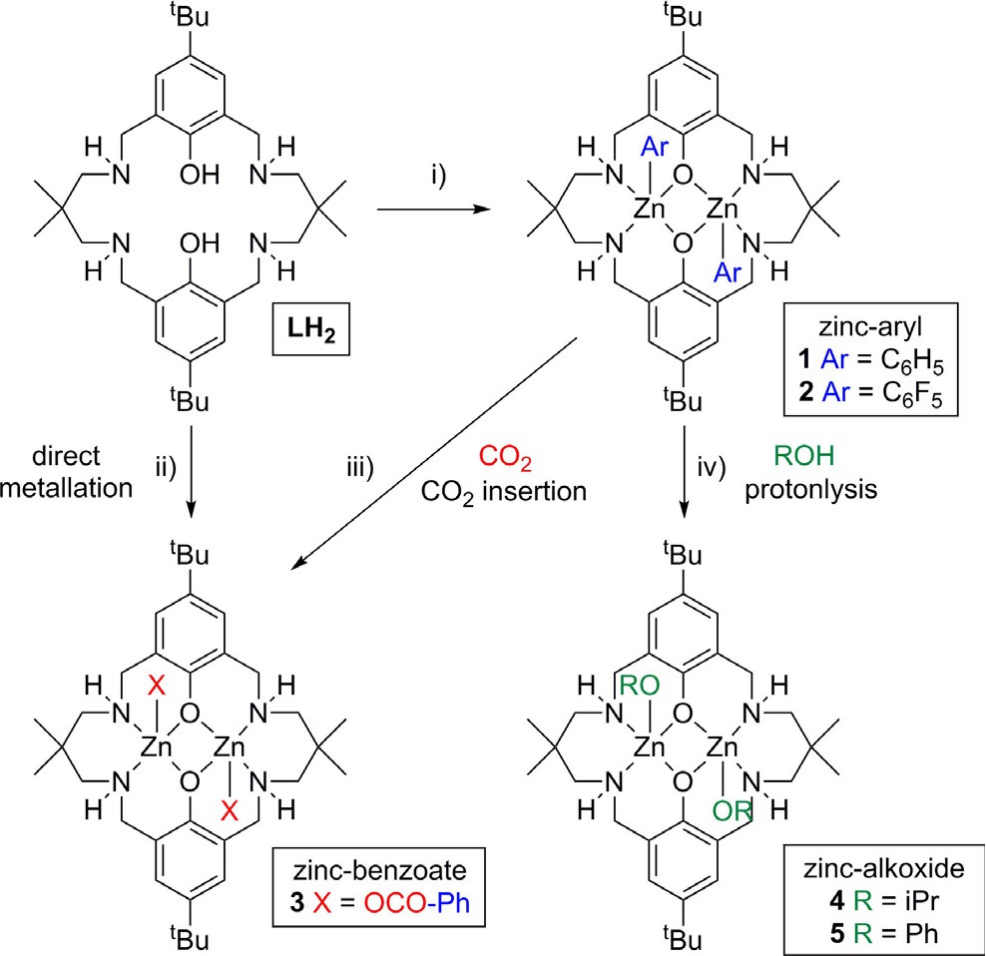
Reactivity overview showing the synthesis of zinc complexes **1**–**5**. Reaction conditions: i) −40 °C to 25 °C, THF solvent, 18 h; **1**, ZnPh_2_ (2 equiv), 81 % crystalline yield; **2**, Zn(C_6_F_5_)_2_ (2 equiv), 52 % crystalline yield; ii) [Zn(OCO‐Ph)_2_] (2 equiv), −40 °C to 25 °C, THF, 18 h, 72 % yield; iii) Starting from **1**, CO_2_ (1 bar), 2 h at 25 °C or 5 minutes at 80 °C; iv) Starting from **1** (1 equiv); **4**, isopropanol (2 equiv), 60 °C, THF, 18 h; **5**, phenol (2 equiv), 25 °C, THF, 18 h, 32 % crystalline yield.

The macrocyclic pro‐ligand **LH_2_** (Scheme 2) was prepared according to literature methods12b and cleanly deprotonated using two equivalents of either ZnPh_2_ or Zn(C_6_F_5_)_2_, in THF at −40 °C to afford the di‐zinc complexes [LZn_2_Ph_2_] (**1**, 81 % yield) and [LZn_2_(C_6_F_5_)_2_] (**2**, 52 % yield), respectively (Scheme 2). For both **1** and **2**, colourless block crystals suitable for X‐ray diffraction studies were obtained by gradual cooling of a hot benzene solution to 25 °C.

**Figure 1:**
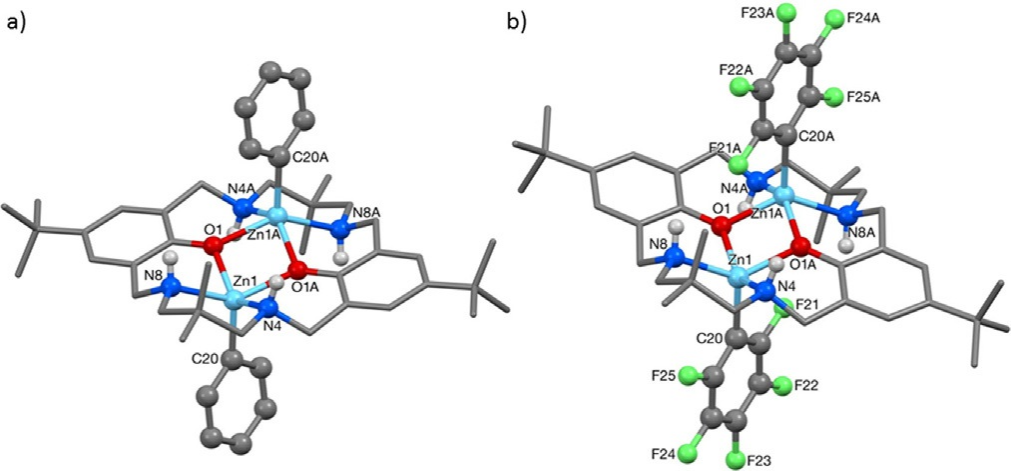
**M**olecular structures of a) [LZn_2_Ph_2_] and b) [LZn_2_(C_6_F_5_)_2_]. Hydrogen atoms and benzene molecules are omitted for clarity.

Structural elucidation by X‐ray diffraction revealed that the two complexes are very similar, and sit across a centre of symmetry at the middle of the Zn_2_O_2_ rings (Figure 1). In contrast to other related di‐Zn complexes based on **LH_2_**, in which the ligand adopts a bowl shape, here the ligand adopts an “S” shape.24b The pentacoordinate Zn atoms, which are bound within the ligand, each share two phenol oxygen atoms. For both **1** and **2**, there is a significant difference between the two different ArO−Zn bond lengths, of 0.13 Å in **1**, and 0.09 Å in **2**. Completing the pentacoordinate geometry, each Zn also bonds to two amine nitrogen atoms and one aryl carbon atom. The aryl‐C−Zn bond lengths lie within the expected range,^26^ although the bond is 0.03 Å shorter in **1** than in **2**. One curious feature is the presence of H−F interactions in **2**, observed between the amine NH and the fluoryl substituents (F21−H8; 2.56(1) Å). Nevertheless, the nature of the co‐ligand does not appear to affect the phenol C−O bond length, which is almost identical within **1** and **2** (O1−C1, 1.341(2) Å and 1.338(2) Å, respectively).

The ^1^H NMR spectrum of **1** is rather complex at room temperature in C_6_D_6_, [D_8_]THF and CDCl_3_. However, at high temperature (403 K in [D_2_]TCE; TCE=1,1,2,2‐tetrachloroethane) an averaged spectrum is obtained consistent with a symmetric structure (Figures S1 and S2). In contrast to **1**, the ^1^H NMR spectrum of **2** shows well‐defined signals at room temperature in CDCl_3_ (Figures S3–S5). For both **1** and **2**, the formation of a dizinc complex was evidenced by four distinct benzylic and methylene resonances. COSY experiments showed that these benzylic and methylene resonances both couple to the N*H* resonance at 2.43 ppm in **1** and at 2.54 ppm in **2**. The ^19^F NMR spectrum of **2** reveals three sharp resonances for the *ortho*, *meta* and *para* resonances, which suggests that the solid‐state H−F interactions are not maintained at 25 °C in solution.

### Reactivity studies

It was of interest to investigate the reactivity of **1** and **2** towards CO_2_, to probe their potential use as polymerisation catalysts.12b, 24 It was observed that **1** reacted with CO_2_ at 1 bar of CO_2_ pressure, in C_6_D_6_ at 25 °C, to afford the corresponding dibenzoate complex [LZn_2_(OCO‐Ph)_2_] (**3)** with complete conversion occurring after two hours, as observed by ^1^H NMR analysis (Scheme 2, Figure S6). The rate of CO_2_ insertion was significantly enhanced by heating the solution to 80 °C, affording complete conversion of **1** to **3**, within 5 min.

**Figure 2:**
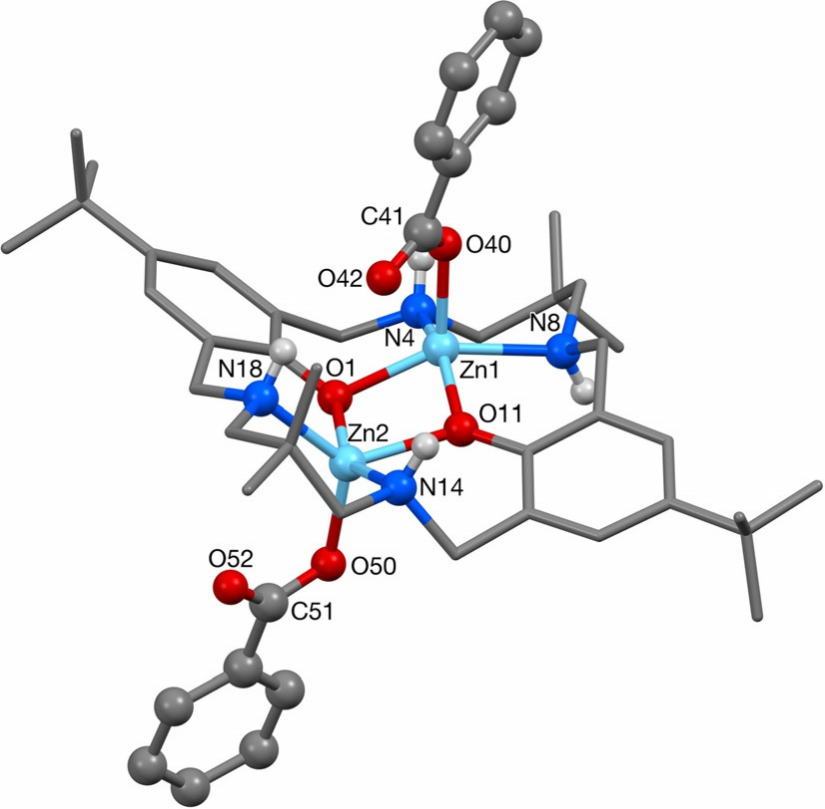
Molecular structure of **3**. Hydrogen atoms and a benzene solvent molecule are omitted for clarity.

To unambiguously confirm the formation of the dibenzoate complex from CO_2_ insertion into **1**, complex **3** was independently synthesised by direct metallation of **LH_2_** by Zn(OCO‐Ph)_2_ at 25 °C in THF (Scheme 2). The ^1^H NMR spectrum was identical to that obtained from CO_2_ insertion into **1**. Colourless block crystals suitable for X‐ray diffraction were obtained at 25 °C from a benzene/THF solvent system, enabling structural elucidation of **3** (Figure 2). The ligand adopts a distorted “S” shape holding two pentacoordinate Zn centres, each with a pendant κ^1^‐*O* benzoate ligand, two bridging phenol O atoms and two secondary amine N atoms. The benzoate C−O bonds differ significantly in length, as the bonds to O52 and O42 are 0.05 Å shorter, suggesting that these contain the most double‐bond character and that O40 and O50 are the anionic donors. At 3.1009(5) Å, the Zn⋅⋅⋅Zn separation is 0.16 Å shorter than that in **1**. This is likely to result from the shortened aryl‐*O*−Zn bonds, which are 0.03 and 0.13 Å shorter than in **1**. Additional, hydrogen bonding interactions between the benzoate O and the amine NH (O42−H18; 2.15(2) Å) provide further stabilisation for **3**.

Complex **3** reproducibly gave rather complex NMR spectra, at high and low temperatures, in a range of different solvents including CDCl_3_, C_6_D_6_ and [D_8_]THF. However, in [D_4_]methanol, a much better resolved ^1^H NMR spectrum was obtained (Figures S7 and S8). The spectrum confirmed the formation of the di‐zinc complex—there are diastereotopic benzylic (4.23 and 3.34 ppm) and methylene (2.91–2.83 ppm) resonances, and the N*H* resonance is observed at 3.15 ppm. It possesses *C*_2_ symmetry in methanol. The benzoate ligands are clearly present as evidenced by the deshielded *ortho*‐phenyl resonance at 7.87 ppm. In the ^13^C NMR spectrum, quaternary carbon resonances were too weak to be observed (including by HMBC experiments) and so a ^13^C‐carbonyl‐labelled sample of **3** was prepared, by the reaction of **1** with two equivalents of ^13^C‐labelled benzoic acid. The carbonyl resonance of **3** is clearly observed at 174.6 ppm, shifted from free benzoic acid (170.1 ppm).

**Figure 3:**
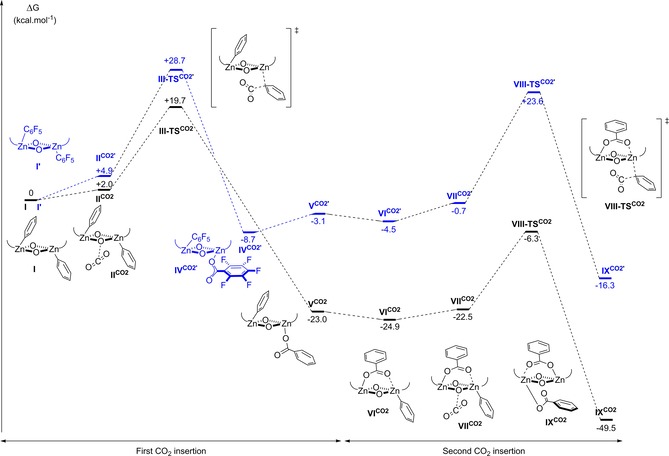
Potential energy surface for the first single CO_2_ insertion into the zinc aryl bond of **1** (black) and **2** (blue); DFT protocol: ωb97xd/6‐31G(d)/cpcm=CH_2_Cl_2_/Temp=353 K. The ancillary ligand structure is omitted for clarity. Interactive version of the figure available at doi.org/10.14469/hpc/2222.

In contrast, the fluoryl analogue, complex **2**, did not react with CO_2_ under identical reaction conditions. It is proposed that the decrease in nucleophilicity of the aryl group, due to the electron‐withdrawing fluoryl substituents, disfavours CO_2_ insertion. This is supported by the observation of a longer, weaker Zn−C bond in **2** (2.049(1) Å) compared to **1** (2.016(1) Å) in the solid‐state crystal structure.^27^ A theoretical study was carried out in order to gain a better understanding of the CO_2_ insertion into the Zn–aryl bonds. DFT was used to calculate the potential energy surface for the stepwise CO_2_ insertion into the Zn–aryl bond for complexes **1** and **2** (Figure 3), to provide insight into the activation energy barriers and the relative stability of the intermediates and products. The calculations were carried out using DFT protocol ωb97xd/6‐31G(d)/srcf(cpcm=dichloromethane) at 353 K, which has previously shown a good agreement with experiments for related reaction studies of similar dinuclear zinc complexes (see the Supporting Information for further details).24b This study focussed on the previously unreported barrier of CO_2_ insertion into the Zn–aryl bond and the calculations reveal the energy barrier to be 9.0 kcal mol^−1^ higher for **I′** (overall barrier Δ*G*=+28.7 kcal mol^−1^) than for **I** (overall barrier Δ*G*=+19.7 kcal mol^−1^). The carbonate products derived from complex **I** are more stable than the corresponding fluoryl analogues obtained from **I′** (ΔΔ*G* up to 20.4 kcal mol^−1^ between **VI** ${}^{CO{}_{2}}$ and **VI** ${}^{CO{}_{2}{}^{'}}$ ), giving further support to the experimental observation that CO_2_ inserts more readily into the Zn−Ph bond than the Zn−C_6_F_5_ analogue.

The calculated mechanism shows CO_2_ insertion occurring at only one metal centre, without participation of the second metal or second aryl co‐ligand (Figure 3). NBO analysis was carried out for **III‐TS** ${}^{CO{}_{2}}$ , which shows a significant interaction between the Zn−C bond and incoming C${}_{\mathrm{CO}{}_{2}}$ atom (see Supporting Information, Figure S9). This contrasts with what was previously observed in the case of a bridging acetate co‐ligand, in which CO_2_ insertion into a Zn–alkoxide bond occurs through a bimetallic mechanism, along with “shuttling” of the electron density of the acetate co‐ligand to balance the charge.24b To allow a comparison between these two systems, the potential energy surface for the second CO_2_ insertion was investigated (Figure 3). Considering the most stable conformation **VI** ${}^{CO{}_{2}}$ , the second CO_2_ insertion into **I** was found to occur by means of a bimetallic mechanism, with nucleophilic attack of the aryl to the CO_2_, and forming a complex with the carboxylate coordinated to one metal centre; concomitantly, the bridging co‐ligand balances the charges. The energy barrier for this second insertion was found to be 18.6 kcal mol^−1^ (between **VI** ${}^{CO{}_{2}}$ and **VIII** ${}^{CO{}_{2}}$ ), which lies close to that determined for the first CO_2_ insertion (19.7 kcal mol^−1^). Overall, the formation of the bis‐carboxylate complex, **IX** ${}^{CO{}_{2}}$ , is highly thermodynamically favoured, with Δ*G*=−49.5 kcal mol^−1^.

Unfortunately, it was not possible to gain experimental evidence for the formation of any intermediates **V** ${}^{CO{}_{2}}$ –**VII** ${}^{CO{}_{2}}$ to confirm this step‐wise model of CO_2_ insertion (i.e., monitoring of the reaction by NMR spectroscopy detected only product **3**). However, it seems reasonable to conclude that the insertion of CO_2_ into the Zn−Ph bond is accessible under the reaction conditions, while the CO_2_ insertion with the fluoryl analogue has a significantly higher energy barrier and is thermodynamically less favoured overall.

**Figure 4:**
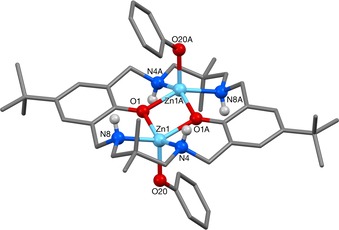
Molecular structure of **5**. Hydrogen atoms and one CH_2_Cl_2_ molecule are omitted for clarity.

A catalyst system, prepared from the in situ reaction of **1** with 1,2‐cyclochexenediol, has previously been applied towards the controlled synthesis of block co‐polymers, through selective catalysis combining the ROP of *ϵ*‐CL with the ROCOP of epoxides and anhydrides.19e However, this catalyst system was prepared and used in situ without detailed characterisation. Thus, it was of interest to investigate the reactivity of **1** and **2** with alcohols (Scheme 1). In these studies, isopropanol was used as a model for the chain‐transfer agent 1,2‐cyclohexenediol. It was selected as a secondary alcohol of similar steric bulk but which simplified spectroscopic characterisation and computational studies compared to 1,2‐cyclohexenediol (vide infra). Although a solution of **1** in THF proved stable in the presence of isopropanol (2 equivalents) at 25 °C, heating the reaction mixture to 60 °C for 18 h led to complete consumption of **1** (Scheme 2). ^1^H NMR analysis, in [D_8_]THF, revealed the formation of a new species, [LZn_2_(O*i*Pr)_2_] (**4**), along with the formation of benzene (singlet at 7.30 ppm) (Figures S10–S12 in the Supporting Information). As the copolymerisations are typically performed at temperatures above 60 °C, this finding suggests that zinc–alkoxide species can form readily under polymerisation conditions.12d In contrast to the broad, convoluted ^1^H NMR spectrum of **1** in [D_8_]THF at 298 K, **4** has a sharp, well‐resolved ^1^H NMR spectrum. Complex **4** was most clearly characterised by the isopropoxide methyne (4.11 ppm) and methyl signals (0.96 ppm), which were shifted compared to free alcohol. Integration of the relevant resonances confirms the 2:1 isopropanol/ligand ratio, showing that complete conversion of both the Zn−Ph bonds to Zn−O*i*Pr groups has occurred. Catalyst **2** also reacted with isopropanol, under reflux conditions in [D_8_]THF; however, a mixture of species was observed, which included **4** and C_6_F_5_H, along with unreacted **2** and *i*PrOH. The reagents **2** and *i*PrOH were still observed after three days at reflux, most likely because the presence of the electron‐withdrawing fluoryl substituents decreases the Brønsted basicity of the phenyl group. Despite several attempts, X‐ray quality crystals of **4** could not be obtained. Instead, the analogous reaction of **1** with phenol (2 equiv) was performed, which led to the formation of the corresponding di‐zinc bis(phenolate) complex [LZn_2_(OPh)_2_] (**5**, Figure 4). Its ^1^H NMR and HSQC experiments reveal the presence of diastereotopic benzylic and methylene protons (Figures S13 and S14).

Crystals of **5** suitable for X‐ray diffraction were crystallised from a mixed THF/CH_2_Cl_2_ solvent system. The molecular structure is centrosymmetric and very similar to **1**, in which the ligand adopts an “S” shaped conformation and both Zn centres are pentacoordinated by the macrocyclic ligand scaffold (two phenol O and two amine N) and a terminal phenol group. There is a significant difference in bond lengths between the bridging and terminal phenols, in which the terminal C‐O‐Zn bond is significantly shorter (by 0.09 Å) than the bridging phenolate bonds from the macrocycle.

**Figure 5:**
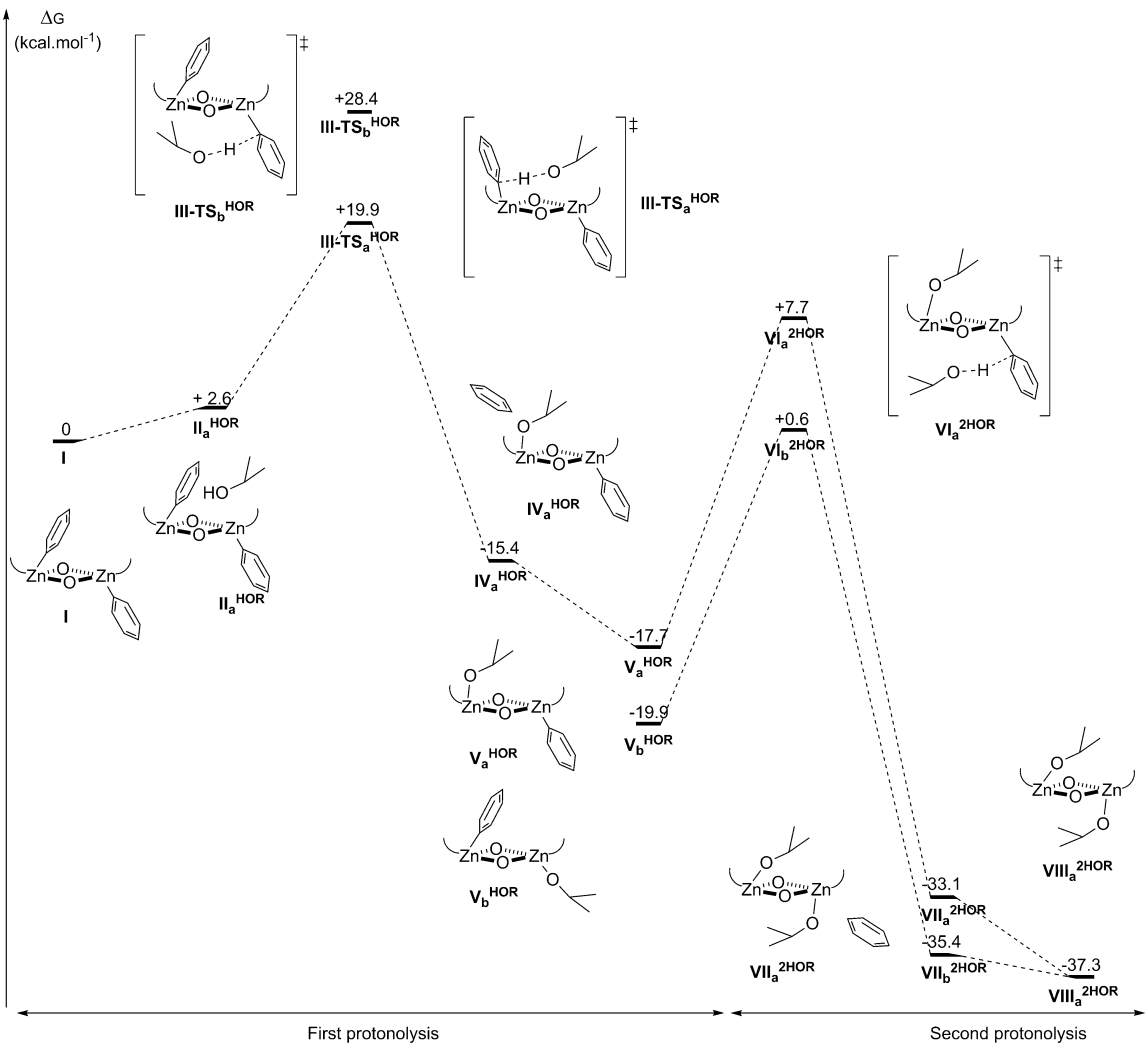
Potential energy surface for the first and second protonolysis of the zinc–aryl bond of **1**, with isopropanol; DFT protocol: ωb97xd/6‐31G(d)/cpcm=CH_2_Cl_2_/Temp=353.15 K (data available at doi.org/10.14469/hpc/2144). The ancillary ligand structure is omitted for clarity.

The reactivity of complex **1** with isopropanol was studied computationally, using CH_2_Cl_2_ as solvent and 353.15 K to mimic polymerisation conditions. The lowest energy pathway was found to have the incoming isopropanol molecule approaching the concave face of the “bowl”‐shaped complex (Figure 5). The energy barrier for the first protonolysis of **1** with isopropanol is +19.9 kcal mol^−1^, which is almost identical to the calculated energy barrier for CO_2_ insertion (+19.7 kcal mol^−1^). The product of the first protonolysis (**V_a_** ^**HOR**^) is thermodynamically favourable (−17.7 kcal mol^−1^). The intermediate can then react with a second equivalent of isopropanol, with an energy barrier of +25.4 kcal mol^−1^, to yield complex **4** (**VIII^2HOR^**), which is calculated to have a relative energy of −37.3 kcal mol^−1^, compared to complex **1** (**I**). This product can also be formed if the protonolysis intermediate (**V_a_** ^**HOR**^) were to undergo a ligand conformational rearrangement, to give a more stable intermediate **V_b_** ^**HOR**^ (by 2.2 kcal mol^−1^). Subsequently, the reaction pathway with isopropanol approaching from the concave face (TS‐**VI_b_** ^**2HOR**^) has a lower energy barrier of +20.5 kcal mol^−1^. The calculations show that the energy barriers for the protonolysis pathways are easily accessible, under polymerisation conditions, and that the products formed are highly stable relative to complex **1**. A key finding is that protonolysis, by reaction with chain‐transfer agents present during polymerisation, is likely to be a highly favourable reaction and that zinc–alkoxide complexes might be considered as the active sites for such catalytic systems.

### Polymerisation studies

Following the successful reaction of **1** with CO_2_, its catalytic activity within CHO/CO_2_ copolymerisation was tested. The polymerisations were run at 0.1 mol % catalyst loading (vs. the epoxide, CHO), using 1 bar of CO_2_ pressure (Table 1, entry 1), as analogous di‐Zn catalysts have previously shown acceptable activity under these conditions.11j The phenyl catalyst **1** is active (TOF=20 h^−1^) and exhibits good CO_2_ uptake, giving >99 % carbonate linkages. The polymerisation is well‐controlled, with a monomodal distribution and a narrow dispersity (1.06). Complex **1** displays similar activity to the previously reported acetate analogue, [LZn_2_(OAc)_2_] (TOF=18 h^−1^, entry 4),12b and significantly outperforms the bromide complex [LZn_2_Br_2_], which is completely inactive under identical reaction conditions.25b Notably, the MALDI‐ToF analysis shows that the purified product is a telechelic polymer terminated by hydroxyl groups (Figure S15), a feature which has been observed with some different catalysts for this copolymerisation.^13^ The formation of dihydroxyl end‐capped polymers is consistent with reactions of [LZn_2_Ph_2_] with alcohol (1,2‐cyclohexenediol) to form the active site.14a The reactivity studies have also demonstrated the capability of **1** to react with CO_2_, within 5 minutes at 80 °C, suggesting that the product di‐zinc–bis(benzoate) complex could initiate copolymerisation. However, benzoate end groups were not observed in the NMR spectroscopy or MALDI‐ToF analysis. Thus it seems likely that the reaction of the zinc–aryl complex with diols, occurs even more rapidly than with CO_2_ and is responsible for the true initiation under these conditions. In line with this observation, catalyst **2** is also active for CHO/CO_2_ ROCOP, in spite of its complete lack of reactivity towards either CHO or CO_2_ in model reactions. Rather **2** is proposed to react with alcohols to generate active alkoxide initiators (Scheme 1). Using catalyst **2**, once again a telechelic polymer is formed, as confirmed by SEC and MALDI‐ToF analysis (Figure S16).14a For both **1** and **2**, the theoretical *M*_n_ values are approximately 12 times greater than the experimental values, which provides further support for the presence of a chain‐transfer agent. The zinc–benzoate analogue, **3**, was also active for CO_2_/epoxide copolymerisation (entry 3) and the MALDI‐ToF analysis of the resultant polymer confirmed the presence of both α‐benzoate, ω‐hydroxy and α,ω‐hydroxy end‐capped polymers (Figure S17). The presence of α‐benzoate end‐groups was confirmed by ^1^H NMR spectroscopy (Figure S18).

**Table 1:** **R**esults for ROP of CHO/CO_2_, PA/CHO and cyclic esters using catalysts **1**, **2**, **3** and [LZn_2_(OAc)_2_].

Entry	Monomer(s)	Cat./Isopropanol/Monomer	*T* [°C]	*t* [h]	TOF^[a]^ [h^−1^]	M_*n*(exp)_^[b]^ [g mol^−1^]	[*Ð*]^[b]^	M_*n*(theo)_ [g mol^−1^]
1	CHO/CO_2_^[c]^	**1**/–/1000	80	20	20	4780	1.06	55 440
2	CHO/CO_2_^[c]^	**2**/–/1000	80	20	20	4280	1.08	56 860
3	CHO/CO_2_^[c]^	**3**/–/1000	80	20	24	6100	1.18	66 810
412b	CHO/CO_2_^[c]^	[LZn_2_(OAc)_2_]/–/1000	80	24	18	6200	1.19	62550
5	PA/CHO^[d]^	**1**/–/100	100	3	33	8620	1.10	12 300^[e]^
6	PA/CHO^[d]^	**2**/–/100	100	3	24	19600	2.27	8870^[e]^
7	PA/CHO^[f]^	**1**/–/200	100	3	21	5610	1.12	7880^[e]^
825a	PA/CHO^[g]^	[LZn_2_(OAc)_2_]/–/100	100	1	24	2570	1.20	2960^[e]^
9	*ϵ*‐CL (tol)^[h]^	**1**/4/500	80	0.08	5280	3900	1.72	13 150
10	*ϵ*‐CL (CH_2_Cl_2_)^[h]^	**1**/4/500	25	2.5	188	4130	1.61	13 410
11	*ϵ*‐CL (CH_2_Cl_2_)^[h]^	[LZn_2_(OAc)_2_]/4/500	25	2.5	0	–	–	–
12	*rac*‐LA (tol)^[h]^	**1**/4/200	80	2	99	7110	1.29	7130
13	*rac*‐LA (tol)^[h]^	[LZn_2_(OAc)_2_]/4/200	80	24	0	–	–	–

[a] Determined by ^1^H NMR spectroscopy. [b] Polymer molecular weights were determined using SEC, calibrated by polystyrene standards, and correction factors were applied as reported previously (1.85 for PA/CHO,19e 0.58 for PLA^29^ or 0.56 for PCL^30^). [c] CO_2_=1 bar. [d] Reaction conditions: 1:100:900 molar ratio of catalyst:PA:CHO. [e] Assuming 2 chains grow per catalyst. [f] Reaction conditions: 1:200:800 molar ratio of catalyst/PA/CHO. [g] Reaction conditions: 1:100:800 molar ratio of catalyst:PA:CHO. [h] Reaction conditions: [M]_0_=1 m.

It has previously been shown that analogues of **1** and **2** with acetate and halide co‐ligands were effective catalysts for the ROCOP of epoxide (CHO)/anhydride (phthalic anhydride PA),^25^ and that when a mixture of monomers is present, anhydride insertion occurs more rapidly than CO_2_ insertion.18a In order to gain further understanding of the polymerisations, complex **1** was tested as a catalyst for the ROCOP of PA/CHO, using a 1 mol % catalyst loading at 100 °C, and neat epoxide as the solvent. After three hours, 100 % conversion was achieved (Table 1, entry 5), with 98 % of alternating enchainment (% ester linkages). The polymerisation is well‐controlled, giving a monomodal MW distribution and a narrow dispersity (*Ð*=1.10). Here, **1** displays a slightly superior activity (TOF=33 h^−1^) compared to its acetate (TOF=24 h^−1^)25a and halide (TOF=17 h^−1^)25b analogues, under analogous conditions. Theoretical calculations suggest that the phenyl co‐ligand could ring‐open PA, as the energy barrier is +29.9 kcal mol^−1^, and the reaction gives a net energy gain of 30.5 kcal mol^−1^ (Figure S19). However, the polyester analysis by MALDI‐ToF again shows only a series of α,ω‐dihydroxyl‐terminated polymers.25a, 28 In this case, the reactivity barrier for insertion of PA into the zinc–phenyl bond is significantly greater than the competing protonolysis pathway, by 10.0 kcal mol^−1^. This suggests that the reaction of complex **1** with alcohols is thermodynamically more favourable than the reaction with PA. It is supported by the absence of phenyl‐capped polymers experimentally. Although **2** displays good activity for CHO/PA ROCOP (TOF=24 h^−1^, entry 6), it is less active than **1** and the polymerisation is poorly controlled, with a broad dispersity (*Ð*=2.27) and low polyester selectivity (23 % polyester vs. polyether). It is therefore observed that the C_6_F_5_ co‐ligand has a detrimental effect, although the exact nature of this influence is not completely clear.

Previously a catalyst system formed in situ by reaction between **1** and 1,2‐cyclohexanediol was investigated for the ROP of *ϵ*‐CL.30a This showed that the catalyst was highly effective, but that polymers with different topologies were formed: indeed, there was evidence for chains both end‐capped by diol and chain extended from the diol. This is because the diol contains two sterically hindered secondary alkoxide groups, which are relatively slower to initiate polymerisations. Given this previous study, it was of interest to study the activation of catalyst **1** with a monofunctional alcohol, so as to ensure that there is only a single type of chain structure. In the presence of isopropanol, **1** was therefore applied to the ROP of *rac*‐lactide and *ϵ*‐caprolactone (Table 1, entries 9–13). Under all conditions tested, **1** demonstrated good catalytic activity under immortal polymerisation conditions (TOF=5280 h^−1^, entry 9). The dispersities are broad, especially for polycaprolactone (PCL). This is attributed to the Zn−Ph reaction with alcohol occurring relatively more slowly at ambient temperature. A series of resonances assigned to α‐isopropoxide, ω‐hydroxy end‐capped polycaprolactone is observed in the MALDI‐ToF spectrum (Figure S20, Table 1, entry 9).

The results show that di‐zinc–aryl **1** can readily react with alcohols, either deliberately added or present as a result of the reaction of water and epoxide, to generate Zn‐alkoxide active sites that can initiate polymerisations. In contrast, the di‐zinc acetate complex, [LZn_2_OAc_2_], does not react with alcohols and lactones and so is not a suitable catalyst for ROP (entry 11 and 13). It is known that the Zn‐carboxylate can react with epoxides to generate Zn‐alkoxide species in situ, which initiate the ROP of lactones, as applied to prepare “switchable” catalysts.18c

Alternatively, the Zn–alkoxide can readily insert CO_2_, whereas its acetate precursor cannot. Although **1** can insert CO_2_ into the Zn−C bond to form a carboxylate, the observation of α,ω‐dihydroxyl end‐capped polymers suggests that reaction of **1** with alcohols occurs more rapidly (Scheme 1). While the acetate catalysts can also undergo chain‐transfer reactions with added alcohols or water,15e, 23a these reactions presumably occur after epoxide opening generates the Zn–alkoxide species.

## Conclusion

In summary, two di‐zinc–aryl complexes have been synthesised from the same macrocyclic ligand and characterised using X‐ray crystallographic and NMR spectroscopic studies. Complex **1** cleanly inserts CO_2_ under mild conditions, whilst **2** is inactive, highlighting differences caused by the electron‐withdrawing fluoryl substituents. The complexes also react readily with alcohols, to generate the di‐zinc–bis(alkoxide) complexes, which were fully characterised. Both **1** and **2** efficiently initiate the alternating copolymerisations of cyclohexene oxide/carbon dioxide and cyclohexene oxide/phthalic anhydride, demonstrating similar activities to the well‐established acetate analogue. The reactivity and theoretical studies suggest that the competing reactions of **1** with CO_2_ or diols are both viable initiation mechanisms for CO_2_/epoxide ROCOP. However, the polymerisation studies suggest that the protonolysis of **1** and **2**, with added or generated alcohols, occurs more rapidly than CO_2_ insertion, and is the predominant initiation mechanism. The in‐situ‐generated alkoxide complex is also an effective catalyst for the ROP of cyclic esters, including both *rac*‐lactide and *ϵ*‐caprolactone, whereas the acetate analogue is completely inactive.

Overall, these studies have led to an improved understanding of the reactivity of di‐zinc–bis(aryl) catalysts, and show how these versatile catalysts can be applied to a range of ROP and ROCOP processes. We expect that the role alcohols can play in initiator formation will facilitate the development of improved future catalyst systems, which will be the focus of our future studies.

## Experimental Section

All metal complexes were synthesised under anhydrous conditions, using MBraun gloveboxes and standard Schlenk techniques. Solvents and reagents were obtained from Sigma Aldrich or Strem and were used as received unless stated otherwise. THF was dried by refluxing over sodium and benzophenone and stored under nitrogen. Isopropanol was dried over calcium hydride and distilled prior to use. Cyclohexene oxide (CHO) was dried over CaH_2_ and fractionally distilled under nitrogen. Phthalic anhydride was purified by dissolving in benzene, filtering off impurities, recrystallising from chloroform and then subliming. All dry solvents and reagents were stored under nitrogen and degassed by several freeze‐pump‐thaw cycles. Research grade carbon dioxide was used for all copolymerisation studies. Macrocyclic ligand **LH_2_** was synthesised following literature procedures.12b NMR spectra spectra were recorded using a Bruker AV 400 MHz spectrometer. Correlations between proton and carbon atoms were obtained by using COSY and HSQC NMR spectroscopic methods. Elemental analysis was determined by Stephen Boyer at London Metropolitan University. SEC was performed using two Mixed Bed PSS SDV linear S columns in series, with THF as the eluent, at a flow rate of 1 mL min^−1^, on a Shimadzu LC‐20AD instrument at 40 °C. Polymer molecular weight (*M*_n_) was determined by comparison against polystyrene standards, with a correction factor of 1.85 for PA/CHO,19e 0.58 for PLA,^29^ and 0.56 for PCL.^30^ The polymer samples were dissolved in SEC grade THF and filtered prior to analysis.

**Crystal structure determination**: Single crystal data were collected using Agilent Xcalibur PX Ultra A (**1** and **2**), Agilent Xcalibur 3 E (**3**) and Oxford Diffraction Xcalibur 3 diffractometers, and the structures were refined using the SHELXTL and SHELX‐2013 program systems.^31^, ^32^ Selected parameters are given in the Supporting Information and full details are given in the deposited cif files. CCDC 1498754 (**1**), 1498755 (**2**), 1498756 (**3**) and 1498757 (**5**) contain the supplementary crystallographic data for this paper. These data are provided free of charge by The Cambridge Crystallographic Data Centre.

### Complex synthesis

**[LZn_2_(Ph)_2_] (1)**: A solution of **H_2_L** (318 mg, 0.57 mmol) in THF (5 mL) was cooled to −40 °C. To this, a pre‐cooled (−40 °C) solution of diphenyl zinc (253 mg, 1.04 mmol) in THF (2 mL) was added. The resultant cloudy solution was stirred overnight at 25 °C, filtered then washed with cold THF (2 mL). The bis‐zinc–phenyl complex **1** was isolated as a white powder (381 mg, 0.46 mmol, 81 % yield). X‐ray quality crystals were obtained by gradual cooling of a hot benzene solution of **1** to 25 °C. ^1^H NMR (CD_2_Cl_4_, 400 MHz, 403 K): *δ*=7.40 (s, 5 H, Ph), 7.00 (s, 4 H, aryl), 4.81 (br m, 4 H, C*H_a_*H_a′_), 3.37 (br d, 4 H, ^2^ *J*_*HaHa′*_=13.3 Hz, CH_a_ *H*_*a′*_), 2.96 (br d, 4 H, ^2^ *J*_*HaHa′*_=10.1 Hz, C*H_b_*H_b′_), 2.89 (br m, 4 H, CH_b_ *H*_*b′*_), 2.47 (br m, 4 H, NH), 1.36 (s, 18 H, *t*Bu), 1.31 (s, 6 H, CH_3_), 1.07 ppm (s, 6 H, CH_3_); ^13^C NMR (CD_2_Cl_4_, 100 MHz, 403 K): *δ*=138.5 (C_quat_, aryl), 128.1 (Ph), 128.0 (C_quat_, aryl), 126.8 (CH, aryl), 123.9 (C_quat_, aryl), 63.5 (CH_2_), 56.7 (CH_2_), 33.4 and 33.3 (C_quat_, *t*Bu and C_quat_, CMe_2_), 31.3 (*t*Bu), 27.9 (CH_3_), 21.5 ppm (CH_3_); elemental analysis calcd (%) for [LZn_2_(Ph)_2_]: C 66.10, H 7.72, N 6.70; found: C 65.98, H 7.77, N 6.68.

**[LZn_2_(C_6_F_5_)_2_] (2)**: To a pre‐cooled (−40 °C) solution of **H_2_L** (200 mg, 0.36 mmol) in THF (5 mL) was added a pre‐cooled (−40 °C) solution of Zn(C_6_F_5_)_2_ (289 mg, 0.72 mmol) in THF (2 mL). After addition, a white suspension started to form. The reaction mixture was allowed to react overnight at 25 °C, and then filtered. The solid product was subsequently washed with cold THF (2 mL) and dried under vacuum to isolate the pure di‐zinc complex **2** as a white powder (180 mg, 0.19 mmol, 52 % yield). ^1^H NMR (CDCl_3_, 400 MHz, 298 K): *δ*=6.75 (s, 4 H, aryl), 4.31 (dd, 4 H, ^2^ *J*_*HaHa′*_=13.3 Hz, ^3^ *J_HaNH_*=12.0 Hz, C*H_a_*H_a′_), 3.33 (d, 4 H, ^2^ *J*_*HaHa′*_=13.3 Hz, CH_a_ *H*_*a′*_), 3.04 (dd, 4 H, ^2^ *J*_*HbHb′*_=12.0 Hz, ^3^ *J_HbNH_=*13.9 Hz, C_*Hb*Hb′_), 2.69 (d, 4 H, ^2^ *J*_*HbHb′*_=12.0 Hz, CH_b_ *H*_*b′*_), 2.54 (dd, 4 H, ^3^ *J_HaNH_*=12.0, ^3^ *J_HbNH_*=14.0 Hz, NH), 1.24 (s, 6 H, CH_3_), 1.21 (s, 18 H, *t*Bu), 1.04 ppm (s, 6 H, CH_3_); ^13^C{H} NMR (CDCl_3_, 100 MHz, 298 K): *δ*=160.6 (C_quat_, aryl), 136.7 (C_quat_, aryl), 126.3 (CH, aryl), 123.3 (C_quat_, aryl), 62.7 (CH_2_), 58.1 (CH_2_), 34.1 and 33.7 (C_quat_, *t*Bu and C_quat_, CMe_2_), 31.7 (CH_3_, *t*Bu), 28.6 (CH_3_), 20.5 ppm (CH_3_); ^19^F NMR (CDCl_3_, 377 MHz, 298 K): *δ*=−115.1 (br s, 2 F), −157.6 (t, 4 F, *J=*20 Hz), −160.8 ppm (m, 4 F); elemental analysis calcd (%) for [LZn_2_(C_6_F_5_)_2_]: C 54.40, H 5.36, N 5.52; found: C 54.25, H 5.45, N 5.39.

**[LZn_2_(OCO‐Ph)_2_] (3)**: To a precooled (−40 °C) solution of **H_2_L** (200 mg, 0.36 mmol) in THF (5 mL) was added a precooled (−40 °C) suspension of [Zn(OCO‐Ph)_2_] (223 mg, 0.72 mmol) in THF (2 mL). A homogeneous solution was produced upon addition, which was stirred overnight at room temperature. After 18 h, a white suspension formed, which was isolated by filtration and washed with hexane (2 mL). The filtrate was cooled down in the freezer (−30 °C) to give a second crop of the white precipitate. Both solids were collected and dried under vacuum to afford **3** as white solid (234 mg, 72 % yield overall). ^1^H NMR ([D_4_]methanol, 400 MHz, 298 K): *δ*=7.88 (br m, 4 H, *o*‐Ph), 7.36 (tt, 2 H, ^3^ *J_HpHm_*=7.6 Hz, ^4^ *J_HpHo_*=1.5 Hz, *p*‐Ph), 7.28 (t, 4 H, ^3^ *J_HmHp_*=7.6 Hz, *m*‐Ph), 6.98 (s, 4 H, aryl), 4.23 (dd, 4 H, ^3^ *J_HaNH_=*12.0 Hz, C*H_a_*H_a′_), 3.34 (d, 4 H, CH_a_ *H*_*a′*_), 3.15 (dd, 4 H, ^3^ *J_NHHa_=*12.0 Hz, NH), 2.91–2.83 (m, 8 H, C_*Hb*Hb“_ and C_*H*bH*b′*_), 1.45 (s, 3 H, CH_3_), 1.21 (s, 6 H, CH_3_), 1.19 (s, 18 H, *t*Bu), 1.04 ppm (s, 3 H, CH_3_); ^13^C{H} NMR ([D_4_]methanol, 100 MHz, 298 K): *δ*=174.64 (C=O),131.9 (*p*‐Ph), 130.6 (*o*‐Ph), 129.3 (CH, aryl), 128.9 (C_quat_), 128.8 (*m*‐Ph), 124.7 (C_quat_), 64.5 (CH_2_), 57.1 (CH_2_), 35.0 (C_quat_), 34.5 (C_quat_), 32.1 (CH_3_, *t*Bu), 32.1 (CH_3_), 28.9 (CH_3_), 21.4 ppm (CH_3_); not all signals for C_quat_ were detected; elemental analysis calcd (%) for [LZn_2_(OCO‐Ph)_2_]: C 62.41, H 6.98, N 6.06; found: C 62.59, H 7.02, N 5.98.

**[LZn_2_(O*i*Pr)_2_] (4)**: NMR scale experiment—in a Youngs tap NMR tube, *i*PrOH (3.7 μL, 48 μmol) was added to a suspension of **1** (20 mg, 24 μmol) in pre‐cooled [D_8_]THF (−40 °C, 0.6 mL). The mixture was allowed to react at room temperature for 15 min then was heated at 60 °C for 18 h to afford a homogeneous solution featuring [LZn_2_(O*i*Pr)_2_] as the major product (along with formation of benzene), as determined by NMR spectroscopy. ^1^H NMR ([D_8_]THF, 400 MHz, 298 K): *δ*=7.30 (s, 12 H, benzene), 6.81 (s, 4 H, aryl), 5.23 (dd, 4 H, ^2^ *J*_*HaHa′*_=12.5 Hz, ^3^ *J_HaNH_*=11.0 Hz, C*H_a_*H_a′_), 4.10 (sept, 2 H, ^3^ *J_HH_*=6.0 Hz, CH O*i*Pr), 3.17 (d, 4 H, ^2^ *J*_*HaHa′*_=12.5 Hz, CH_a_ *H*_*a′*_), 2.89 (dd, 4 H, ^2^ *J*_*HbHb′*_=13.3 Hz, ^3^ *J_HbNH_*=13.3 Hz, C_*Hb*Hb′_), 2.60–2.57 (m, 8 H, CH_2_ and NH), 1.28–1.22 (m, 40 H, *t*Bu and CH_3_), 0.96 ppm (d, 12 H, ^3^J_HH_=6 Hz, CH_3_ O*i*Pr); ^13^C{H} NMR ([D_8_]THF, 100 MHz, 298 K): *δ*=161.7 (C_quat_, aryl), 136.1 (C_quat_, aryl), 129.2 (benzene), 126.9 (CH, aryl), 125.5 (C_quat_, aryl), 66.5 (CH, O*i*Pr), 63.7 (CH_2_), 58.1 (CH_2_), 34.5 (C_quat_, *t*Bu), 34.2 (C_quat_, *t*Bu), 32.3 (CH_3_, *t*Bu), 31.0 (CH_3_, O*i*Pr), 28.8 (CH_3_), 21.0 ppm (CH_3_).

**[LZn_2_(PhO)_2_] (5)**: Complex **1** was prepared on a 0.24 mmol scale, following the procedure described above. Phenol (45 mg, 0.48 mmol) was added in situ and the reaction mixture was allowed to stir for 18 h. The resultant white solid was isolated via filtration and was dried under vacuum, to give **5** as a white powder (66 mg, 32 % yield). ^1^H NMR ([D_8_]THF, 500 MHz, 328 K): *δ*=6.78 (s, 4 H, aryl), 6.70 (dd, 4 H, ^3^ *J_HmHo_*=8.5 Hz, ^3^ *J_HmHp_*=7.8 Hz, *m*‐Ph), 6.55 (d, 4 H, ^3^ *J_HoHm_*=8.5 Hz, ^4^ *J_HoHp_*=1.0 Hz, *o*‐Ph), 6.09 (t, 2 H, ^3^ *J_HpHm_*=7.3 Hz, ^4^ *J_HpHo_*=1.0 Hz, *p*‐Ph), 4.99 (dd, 4 H, ^2^ *J*_*HaHa′*_ *=*12.9 Hz, ^3^ *J_HaNH_=*11.1 Hz, C*H_a_*H_a′_), 3.24 (d, 4 H, ^2^ *J*_*HaHa′*_=12.9 Hz, CH_a_ *H*_*a′*_), 3.07 (dd, 4 H, ^3^ *J_NHHb_*=13.6 Hz, ^3^ *J_NHHa_=*11.1 Hz, ^3^ *J*_*NHHb′*_=3.6 Hz, NH), 2.98 (dd, 4 H, ^2^ *J*_*HbHb′*_=10.6 Hz, ^3^ *J_HbNH_*=13.6 Hz, C_*Hb*Hb′_), 2.71 (dd, 4 H, ^2^ *J*_*HbHb′*_=10.6 Hz, ^3^ *J*_*Hb′NH*_=3.6 Hz, C_Hb*Hb′*_), 1.27 (br m, 12 H, *t*Bu and CH_3_), 1.23 (s, 3 H, CH_3_), 1.18 (s, 9 H, *t*Bu), 1.16 (s, 3 H, CH_3_), 0.98 ppm (s, 3 H, CH_3_); residual CH_2_Cl_2_ at 5.46 ppm; ^13^C{H} NMR ([D_8_]THF, 126 MHz, 328 K): *δ*=129.2 (*m*‐Ph), 127.0 (CH aryl), 119.7 (*o*‐Ph), 112.7 (*p*‐Ph), 63.9 (CH_2_), 57.8 (CH_2_), 32.1 (CH_3_), 32.0 (CH_3_), 32.0 (CH_3_, *t*Bu), 28.0 (CH_3_), 21.1 ppm (CH_3_); signals for C_quat_ were not detected; elemental analysis calcd (%) for [LZn_2_(PhO)_2_]: C 63.67, H 7.43, N 6.46; found: C 63.54, H 7.53, N 6.55.

## Conflict of interest

Charlotte Williams is a founder and director of econic technologies.

## Supporting information

As a service to our authors and readers, this journal provides supporting information supplied by the authors. Such materials are peer reviewed and may be re‐organized for online delivery, but are not copy‐edited or typeset. Technical support issues arising from supporting information (other than missing files) should be addressed to the authors.

Supplementary

## References

[1a] D. Seyferth , Organometallics 2001, 20, 2940–2955;

[1b] E. Frankland , J. Chem. Soc. 1850, 2, 263–296.

[2a] A. O. King , N. Okukado , E.-i. Negishi , J. Chem. Soc. Chem. Commun. 1977, 683–684;

[2b] E.-i. Negishi , T. Takahashi , A. O. King , Org. Synth. 1988, 66, 67–72.

[3] F. F. Kneisel , M. Dochnahl , P. Knochel , Angew. Chem. Int. Ed. 2004, 43, 1017–1021; 10.1002/anie.200353316 14966896

[4a] K. Yearick , C. Wolf , Org. Lett. 2008, 10, 3915–3918; 18686960 10.1021/ol8015012

[4b] E. Hevia , A. R. Kennedy , J. Klett , Z. Livingstone , M. D. McCall , Dalton Trans. 2010, 39, 520–526. 10.1039/b911818g 20023989

[5a] J. Lewiński , Z. Ochal , E. Bojarski , E. Tratkiewicz , I. Justyniak , J. Lipkowski , Angew. Chem. Int. Ed. 2003, 42, 4643–4646; 10.1002/anie.200351940 14533152

[5b] M. Kubisiak , K. Zelga , I. Justyniak , E. Tratkiewicz , T. Pietrzak , A. R. Keeri , Z. Ochal , L. Hartenstein , P. W. Roesky , J. Lewiński , Organometallics 2013, 32, 5263–5265.

[6] D. Łowicki , S. Baś , J. Mlynarski , Tetrahedron 2015, 71, 1339–1394.

[7a] M. Cheng , A. B. Attygalle , E. B. Lobkovsky , G. W. Coates , J. Am. Chem. Soc. 1999, 121, 11583–11584;

[7b] B. M. Chamberlain , M. Cheng , D. R. Moore , T. M. Ovitt , E. B. Lobkovsky , G. W. Coates , J. Am. Chem. Soc. 2001, 123, 3229–3238; 11457057 10.1021/ja003851f

[7c] C. K. Williams , L. E. Breyfogle , S. K. Choi , W. Nam , V. G. Young , M. A. Hillmyer , W. B. Tolman , J. Am. Chem. Soc. 2003, 125, 11350–11359; 16220958 10.1021/ja0359512

[7d] B. Gao , R. Duan , X. Pang , X. Li , Z. Qu , H. Shao , X. Wang , X. Chen , Dalton Trans. 2013, 42, 16334–16342; 24065120 10.1039/c3dt52016a

[7e] M. H. Chisholm , N. W. Eilerts , J. C. Huffman , S. S. Iyer , M. Pacold , K. Phomphrai , J. Am. Chem. Soc. 2000, 122, 11845–11854;

[7f] H.-Y. Chen , B.-H. Huang , C.-C. Lin , Macromolecules 2005, 38, 5400–5405.

[8a] H. S. Kim , J. Y. Bae , J. S. Lee , O. S. Kwon , P. Jelliarko , S. D. Lee , S.-H. Lee , J. Catal. 2005, 232, 80–84;

[8b] M. Ramin , J.-D. Grunwaldt , A. Baiker , J. Catal. 2005, 234, 256–267;

[8c] D. Anselmo , V. Bocokić , A. Decortes , E. C. Escudero-Adán , J. Bene tBuchholz, J. N. H. Reek, A. W. Kleij, Polyhedron **2012**, 32, 49–53.

[9a] B. M. Trost , H. Ito , J. Am. Chem. Soc. 2000, 122, 12003–12004;

[9b] B. M. Trost , E. R. Silcoff , H. Ito , Org. Lett. 2001, 3, 2497–2500. 11483044 10.1021/ol0161211

[10a] A. Zulys , M. Dochnahl , D. Hollmann , K. Löhnwitz , J.-S. Herrmann , P. W. Roesky , S. Blechert , Angew. Chem. Int. Ed. 2005, 44, 7794–7798; 10.1002/anie.200502006 16270367

[10b] M. Dochnahl , J.-W. Pissarek , S. Blechert , K. Lohnwitz , P. W. Roesky , Chem. Commun. 2006, 3405–3407. 10.1039/b607597e 16896476

[11a] D. J. Darensbourg , M. W. Holtcamp , Coord. Chem. Rev. 1996, 153, 155–174;

[11b] D. R. Moore , M. Cheng , E. B. Lobkovsky , G. W. Coates , J. Am. Chem. Soc. 2003, 125, 11911–11924; 14505413 10.1021/ja030085e

[11c] D. J. Darensbourg , Chem. Rev. 2007, 107, 2388–2410; 17447821 10.1021/cr068363q

[11d] G. A. Luinstra , Polym. Rev. 2008, 48, 192–219;

[11e] S. I. Vagin , R. Reichardt , S. Klaus , B. Rieger , J. Am. Chem. Soc. 2010, 132, 14367–14369; 20863071 10.1021/ja106484t

[11f] S. Klaus , M. W. Lehenmeier , E. Herdtweck , P. Deglmann , A. K. Ott , B. Rieger , J. Am. Chem. Soc. 2011, 133, 13151–13161; 21744837 10.1021/ja204481w

[11g] G.-P. Wu , D. J. Darensbourg , X.-B. Lu , J. Am. Chem. Soc. 2012, 134, 17739–17745; 23016983 10.1021/ja307976c

[11h] D. J. Darensbourg , S. J. Wilson , Green Chem. 2012, 14, 2665–2671;

[11i] Y. Liu , W.-M. Ren , J. Liu , X.-B. Lu , Angew. Chem. Int. Ed. 2013, 52, 11594–11598; 10.1002/anie.201305154 24019292

[11j] M. I. Childers , J. M. Longo , N. J. Van Zee , A. M. LaPointe , G. W. Coates , Chem. Rev. 2014, 114, 8129–8152; 25007101 10.1021/cr400725x

[11k] W. C. Ellis , Y. Jung , M. Mulzer , R. Di Girolamo , E. B. Lobkovsky , G. W. Coates , Chem. Sci. 2014, 5, 4004–4011;

[11l] Y. Liu , W.-M. Ren , K.-K. He , X.-B. Lu , Nat. Commun. 2014, 5, 5687; 25477252 10.1038/ncomms6687

[11m] S. Kissling , P. T. Altenbuchner , M. W. Lehenmeier , E. Herdtweck , P. Deglmann , U. B. Seemann , B. Rieger , Chem. Eur. J. 2015, 21, 8148–8157; 25900151 10.1002/chem.201406055

[11n] S. Paul , Y. Zhu , C. Romain , R. Brooks , P. K. Saini , C. K. Williams , Chem. Commun. 2015, 51, 6459–6479; 10.1039/c4cc10113h 25688813

[11o] M. Mandal , D. Chakraborty , J. Polym. Sci. Part A 2016, 54, 809–824.

[12a] D. R. Moore , M. Cheng , E. B. Lobkovsky , G. W. Coates , Angew. Chem. Int. Ed. 2002, 41, 2599–2602; 10.1002/1521-3773(20020715)41:14<2599::AID-ANIE2599>3.0.CO;2-N 12203547

[12b] M. R. Kember , P. D. Knight , P. T. R. Reung , C. K. Williams , Angew. Chem. Int. Ed. 2009, 48, 931–933; 10.1002/anie.200803896 19115338

[12c] M. R. Kember , A. J. P. White , C. K. Williams , Inorg. Chem. 2009, 48, 9535–9542; 19780624 10.1021/ic901109e

[12d] M. R. Kember , A. Buchard , C. K. Williams , Chem. Commun. 2011, 47, 141–163; 10.1039/c0cc02207a 20941400

[12e] M. W. Lehenmeier , S. Kissling , P. T. Altenbuchner , C. Bruckmeier , P. Deglmann , A.-K. Brym , B. Rieger , Angew. Chem. Int. Ed. 2013, 52, 9821–9826; 10.1002/anie.201302157 23873829

[13a] H. Sugimoto , K. Kuroda , Macromolecules 2008, 41, 312–317;

[13b] W. J. van Meerendonk , R. Duchateau , C. E. Koning , G.-J. M. Gruter , Macromolecules 2005, 38, 7306–7313;

[13c] Y. Xiao , Z. Wang , K. Ding , Chem. Eur. J. 2005, 11, 3668–3678; 15827981 10.1002/chem.200401159

[13d] M. R. Kember , A. J. P. White , C. K. Williams , Macromolecules 2010, 43, 2291–2298.

[14a] G.-P. Wu , D. J. Darensbourg , Macromolecules 2016, 49, 807–814;

[14b] Y. Wang , J. Fan , D. J. Darensbourg , Angew. Chem. Int. Ed. 2015, 54, 10206–10210; 10.1002/anie.201505076 26177634

[14c] D. J. Darensbourg , G.-P. Wu , Angew. Chem. Int. Ed. 2013, 52, 10602–10606; 10.1002/anie.201304778 23946153

[15a] M. Peters , B. Köhler , W. Kuckshinrichs , W. Leitner , P. Markewitz , T. E. Müller , ChemSusChem 2011, 4, 1216–1240; 21866580 10.1002/cssc.201000447

[15b] A. Cyriac , S. H. Lee , J. K. Varghese , J. H. Park , J. Y. Jeon , S. J. Kim , B. Y. Lee , Green Chem. 2011, 13, 3469–3475;

[15c] S. H. Lee , A. Cyriac , J. Y. Jeon , B. Y. Lee , Polym. Chem. 2012, 3, 1215–1220;

[15d] N. von der Assen , A. Bardow , Green Chem. 2014, 16, 3272–3280;

[15e] A. M. Chapman , C. Keyworth , M. R. Kember , A. J. J. Lennox , C. K. Williams , ACS Catal. 2015, 5, 1581–1588.

[16] C. D. Cowman , E. Padgett , K. W. Tan , R. Hovden , Y. Gu , N. Andrejevic , D. Muller , G. W. Coates , U. Wiesner , J. Am. Chem. Soc. 2015, 137, 6026–6033. 25836760 10.1021/jacs.5b01915 PMC4434530

[17] M. H. Chisholm , Z. Zhou , J. Mater. Chem. 2004, 14, 3081–3092.

[18a] C. Romain , Y. Zhu , P. Dingwall , S. Paul , H. S. Rzepa , A. Buchard , C. K. Williams , J. Am. Chem. Soc. 2016, 138, 4120–4131; 27003333 10.1021/jacs.5b13070

[18b] C. Romain , A. Thevenon , P. K. Saini , C. K. Williams , in Carbon Dioxide and Organometallics (Ed.: X.-B. Lu), Springer, Cham (Germany), 2015, pp. 101–141;

[18c] C. Romain , C. K. Williams , Angew. Chem. Int. Ed. 2014, 53, 1607–1610; 10.1002/anie.201309575 PMC4232277 24453135

[19a] M. Cheng , E. B. Lobkovsky , G. W. Coates , J. Am. Chem. Soc. 1998, 120, 11018–11019;

[19b] M. Cheng , D. R. Moore , J. J. Reczek , B. M. Chamberlain , E. B. Lobkovsky , G. W. Coates , J. Am. Chem. Soc. 2001, 123, 8738–8749; 11535078 10.1021/ja003850n

[19c] S. D. Allen , D. R. Moore , E. B. Lobkovsky , G. W. Coates , J. Am. Chem. Soc. 2002, 124, 14284–14285; 12452684 10.1021/ja028071g

[19d] M. F. Pilz , C. Limberg , B. B. Lazarov , K. C. Hultzsch , B. Ziemer , Organometallics 2007, 26, 3668–3676;

[19e] Y. Zhu , C. Romain , C. K. Williams , J. Am. Chem. Soc. 2015, 137, 12179–12182. 26374097 10.1021/jacs.5b04541

[20] C. Romain , M. S. Bennington , A. J. P. White , C. K. Williams , S. Brooker , Inorg. Chem. 2015, 54, 11842–11851. 26624788 10.1021/acs.inorgchem.5b02038

[21a] S. Inoue , Y. Yokoo , J. Organomet. Chem. 1972, 39, 11–16;

[21b] S. Schulz , S. Schmidt , D. Bläser , C. Wölper , Eur. J. Inorg. Chem. 2011, 4157–4160;

[21c] D. Specklin, C. Fliedel, C. Gourlaouen, J.-C. Bruyere, T. Avilés, C. Boudon, L. Ruhlmann, S. Dagorne, *Chem. Eur. J* **2017**, https://doi.org/10.1002/chem.201605907. 10.1002/chem.201605907 28220966

[22] N. J. Brown , J. E. Harris , X. Yin , I. Silverwood , A. J. P. White , S. G. Kazarian , K. Hellgardt , M. S. P. Shaffer , C. K. Williams , Organometallics 2014, 33, 1112–1119. 24882918 10.1021/om400679n PMC4034080

[23a] M. R. Kember , C. K. Williams , J. Am. Chem. Soc. 2012, 134, 15676–15679; 22971183 10.1021/ja307096m

[23b] A. Buchard , M. R. Kember , K. G. Sandeman , C. K. Williams , Chem. Commun. 2011, 47, 212–214. 10.1039/c0cc02205e 20871911

[24a] F. Jutz , A. Buchard , M. R. Kember , S. B. Fredriksen , C. K. Williams , J. Am. Chem. Soc. 2011, 133, 17395–17405; 21973120 10.1021/ja206352x

[24b] A. Buchard , F. Jutz , M. R. Kember , A. J. P. White , H. S. Rzepa , C. K. Williams , Macromolecules 2012, 45, 6781–6795.

[25a] P. K. Saini , C. Romain , Y. Zhu , C. K. Williams , Polym. Chem. 2014, 5, 6068–6075;

[25b] J. A. Garden , P. K. Saini , C. K. Williams , J. Am. Chem. Soc. 2015, 137, 15078–15081; 26618526 10.1021/jacs.5b09913

[25c] M. Winkler , C. Romain , M. A. R. Meier , C. K. Williams , Green Chem. 2015, 17, 300–306.

[26a] P. R. Markies , G. Schat , O. S. Akkerman , F. Bickelhaupt , W. J. J. Smeets , A. L. Spek , Organometallics 1990, 9, 2243–2247;

[26b] P. R. Markies , G. Schat , O. S. Akkerman , F. Bickelhaupt , A. L. Spek , J. Organomet. Chem. 1992, 430, 1–13;

[26c] R. S. Dickson , G. D. Fallon , Q.-Q. Zhang , J. Chem. Soc. Dalton Trans. 2000, 1973–1974;

[26d] P. C. Andrikopoulos , D. R. Armstrong , H. R. L. Barley , W. Clegg , S. H. Dale , E. Hevia , G. W. Honeyman , A. R. Kennedy , R. E. Mulvey , J. Am. Chem. Soc. 2005, 127, 6184–6185; 15853319 10.1021/ja050860l

[26e] A. Lennartson , M. Hakansson , Acta Crystallogr. Sect. C 2009, 65, m205–m207; 10.1107/S0108270109011469 19407405

[26f] A. Hernán-Gómez , E. Herd , M. Uzelac , T. Cadenbach , A. R. Kennedy , I. Borilovic , G. Aromí , E. Hevia , Organometallics 2015, 34, 2614–2623.

[27] K. Nakano , K. Nozaki , T. Hiyama , J. Am. Chem. Soc. 2003, 125, 5501–5510. 12720465 10.1021/ja028666b

[28] E. H. Nejad , A. Paoniasari , C. G. W. van Melis , C. E. Koning , R. Duchateau , Macromolecules 2013, 46, 631–637.

[29a] C. Bakewell , A. J. P. White , N. J. Long , C. K. Williams , Angew. Chem. Int. Ed. 2014, 53, 9226–9230; 10.1002/anie.201403643 PMC4499264 25044165

[29b] A. Kowalski , A. Duda , S. Penczek , Macromolecules 1998, 31, 2114–2122.

[30a] Y. Zhu , C. Romain , V. Poirier , C. K. Williams , Macromolecules 2015, 48, 2407–2416;

[30b] M. Save , M. Schappacher , A. Soum , Macromol. Chem. Phys. 2002, 203, 889–899.

[31] SHELXTL v5.1, Bruker AXS, Madison, WI, **1998**.

[32] SHELX-2013, G. M. Sheldrick, Acta Crystallogr. Sect. C **2015**, *71*, 3–8.

