# Supplementary material for: Di‐Zinc–Aryl Complexes: CO2 Insertions and Applications in Polymerisation Catalysis
Source: Chemistry. 2017 May 5;23(30):7367–76. doi: 10.1002/chem.201701013 (PMC5488170; doi:10.1002/chem.201701013)
Supplement: Supplementary file 1 — Supplementary [file CHEM-23-7367-s001.pdf]

CHEMISTRY
A European Journal

Supporting Information

Di-Zinc–Aryl Complexes: CO_2 Insertions and Applications in Polymerisation Catalysis

Charles Romain+,[b] Jennifer A. Garden+,[c] Gemma Trott,[a] Antoine Buchard,[d] Andrew J. P. White,[b] and Charlotte K. Williams*[a, b]

chem_201701013_sm_miscellaneous_information.pdf

Author Contributions

C.R. Investigation: Equal
J.G. Investigation: Equal
G.T. Investigation: Supporting
A.B. Investigation: Supporting
A.W. Investigation: Supporting.

Supporting Information

Table of Contents

Page S2: ^{1}H NMR of **1** in d_2-TCE solvent at 403 K (**Figure S1**)

Page S2: ^{13}C NMR of **1** in d_2-TCE solvent at 403 K (**Figure S2**)

Page S3: ^{1}H NMR of **2** in $CDCl_3$ solvent at 298 K (**Figure S3**)

Page S3: ^{13}C NMR of **2** in $CDCl_3$ solvent at 298 K (**Figure S4**)

Page S4: ^{19}F NMR of **2** in $CDCl_3$ solvent at 298 K (**Figure S5**)

Page S4: Overlay of ^{1}H NMR spectra of complex **1** with and without CO_2 insertion (**Figure S6**)

Page S5: ^{1}H NMR of **3** in d_4–methanol solvent at 298 K (**Figure S7**)

Page S5: ^{13}C NMR of **3** in d_4–methanol solvent at 298 K (**Figure S8**)

Page S6: Discussion of DFT Calculations and NBO analysis

Page S7: NBO analysis of **III-TS^{CO2}** (**Figure S9**)

Page S8: ^{1}H NMR of **4** in d_8-THF solvent at 298 K (**Figure S10**)

Page S8: ^{13}C NMR of **4** in d_8-THF solvent at 298 K (**Figure S11**)

Page S9: Overlay of ^{1}H NMR spectra showing reaction of **1** with ^{i}PrOH (**Figure S12**)

Page S9: ^{1}H NMR of **5** in d_8-THF solvent at 328 K (**Figure S13**)

Page S10: HSQC of **5** in d_8-THF solvent at 328 K (**Figure S14**)

Page S10: MALDI-ToF mass spectra of PCHC produced with complex **1** (**Figure S15**)

Page S11: MALDI-ToF mass spectra of PCHC produced with complex **2** (**Figure S16**)

Page S11: MALDI-ToF mass spectra of PCHC produced with complex **3** (**Figure S17**)

Page S12: ^{1}H NMR spectrum of crude PCHC showing benzoate initiating groups from **3** (**Figure S18**)

Page S12: Potential energy surface for ring opening of PA by the Zn-aryl bond of **1** (**Figure S19**)

Page S13: MALDI-ToF mass spectra of polycaprolactone produced with complex **1** (**Figure S20**)

Page S14: CHO-CO_2 Copolymerisation Reactions (**Figures S21-S22**)

Page S15: CHO-PA Copolymerisation Reactions (**Figures S23-S24**)

Page S16: ε-Caprolactone Polymerisation Reactions (**Figure S25-S26**)

Page S17: rac-Lactide Polymerisation Reactions (**Figures S27-S29**)

Page S19: X-Ray Crystallography Data (**Tables S1-S2**; **Figures S30-S33**)

Page S24: References

Interactive and complementary data available free of charge from the Imperial College High Performance Computing Service Data Repository at doi.org/10.14469/hpc/2144.

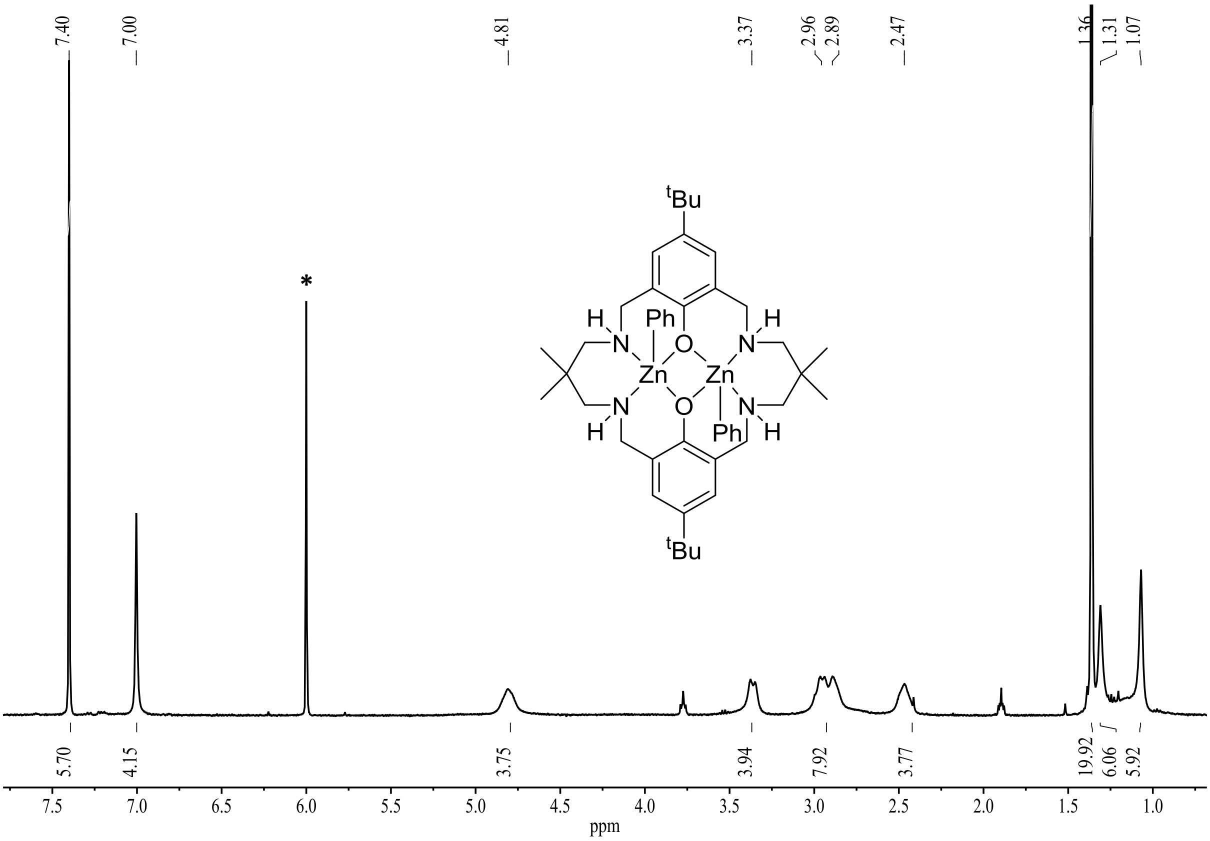

Figure S1. ^{1}H NMR of **1** in d_2-TCE solvent at 403 K. Free benzene was observed due to the reaction of **1** with traces of water in the d_2-TCE solvent (data available at 10.14469/hpc/2151).

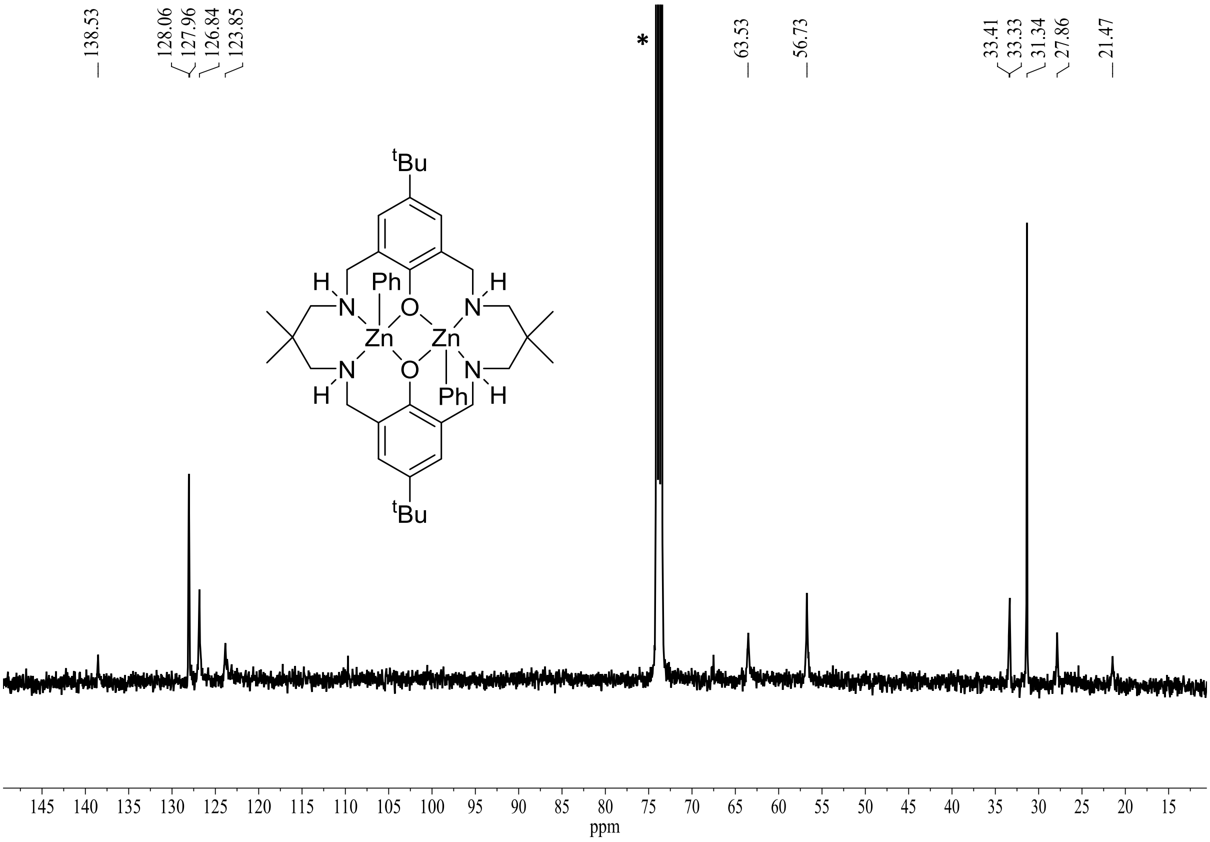

Figure S2. ^{13}C NMR of **1** in d_2-TCE solvent at 403 K (data available at 10.14469/hpc/2151).

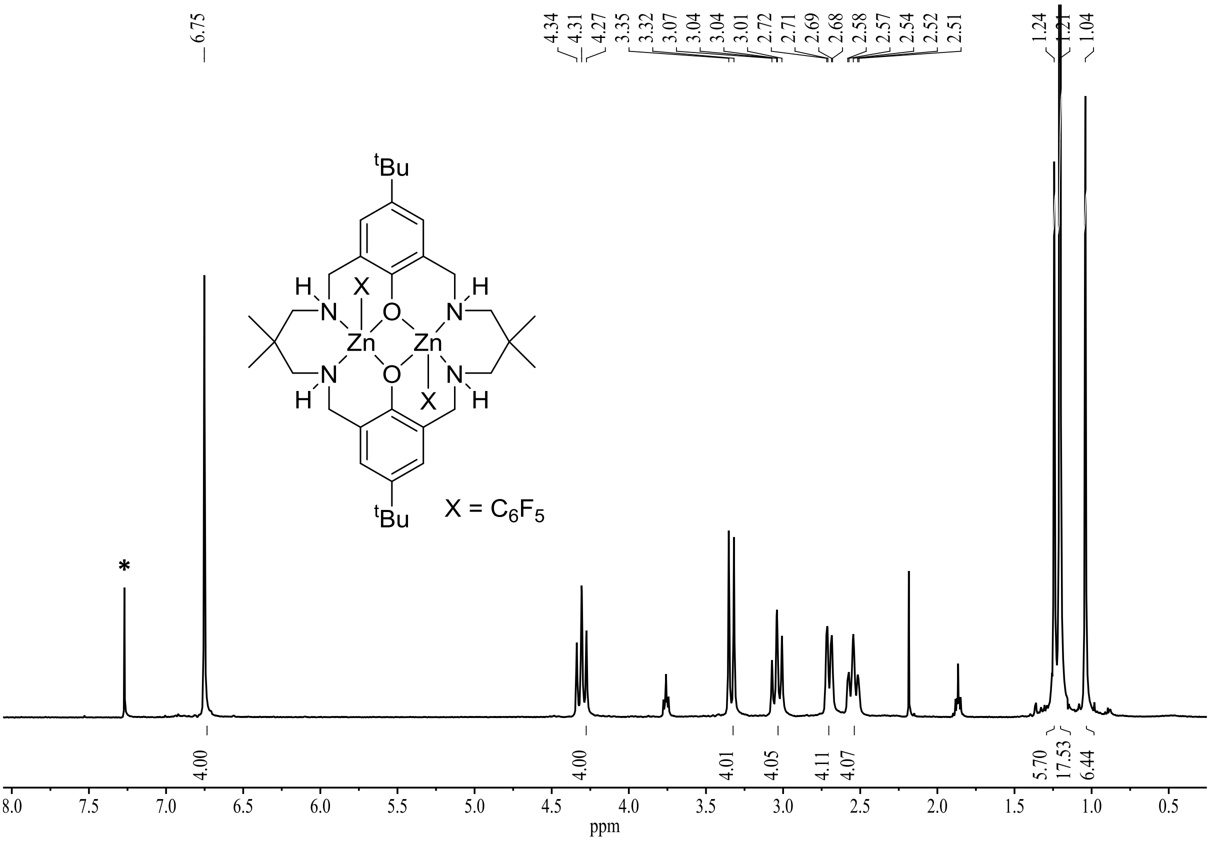

Figure S3. ^{1}H NMR of **2** in $CDCl_3$ solvent at 298 K (data available at 10.14469/hpc/2151).

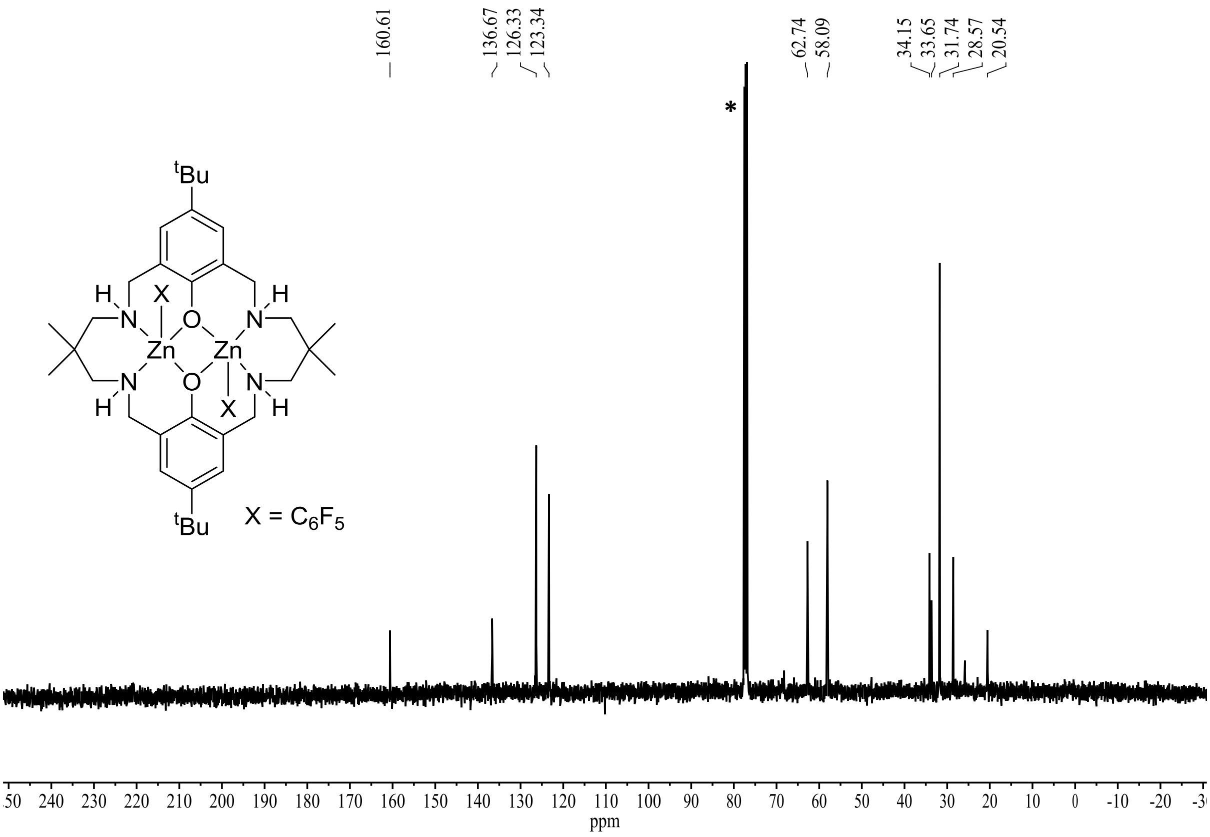

Figure S4. ^{13}C NMR of **2** in $CDCl_3$ solvent at 298 K (data available at 10.14469/hpc/2151).

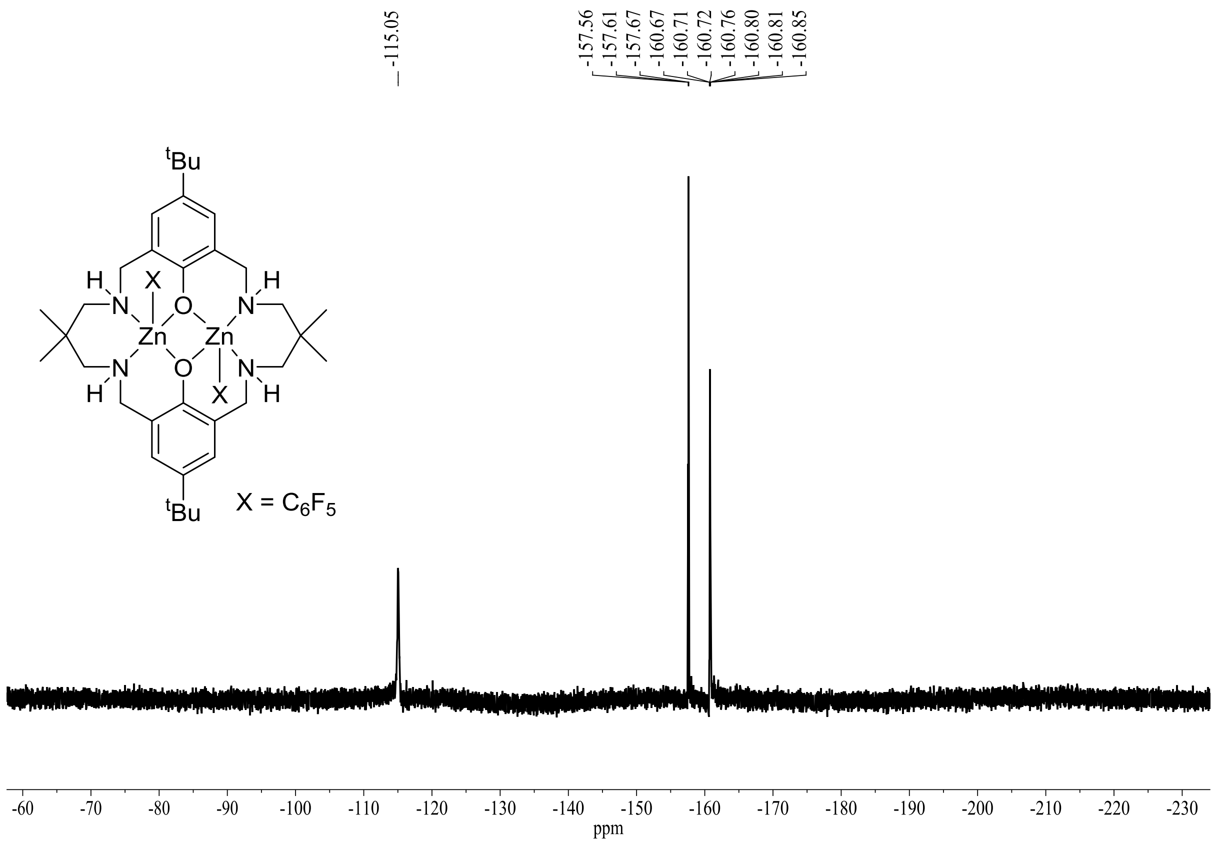

Figure S5. ^{19}F NMR of **2** in $CDCl_3$ solvent at 298 K (data available at 10.14469/hpc/2151).

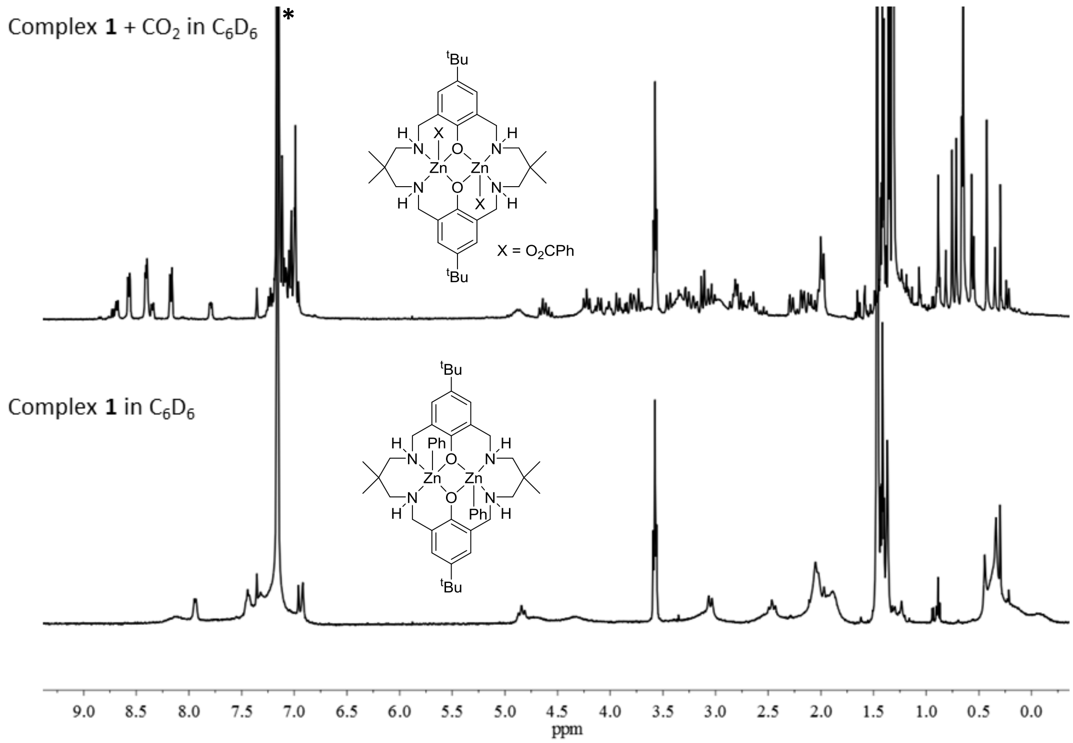

Figure S6. Overlay of ^{1}H NMR spectra of complex **1** (bottom) and complex **1** with CO_2 insertion (top), in C_6D_6 solvent at 298 K (data available at 10.14469/hpc/2151). The top spectrum is identical to that of crystalline **3** in C_6D_6 solvent. Complete disappearance of the aromatic resonance at 7.94 ppm is observed upon CO_2 insertion, and new aromatic resonances are observed (8.57, 8.41 and 8.17 ppm).

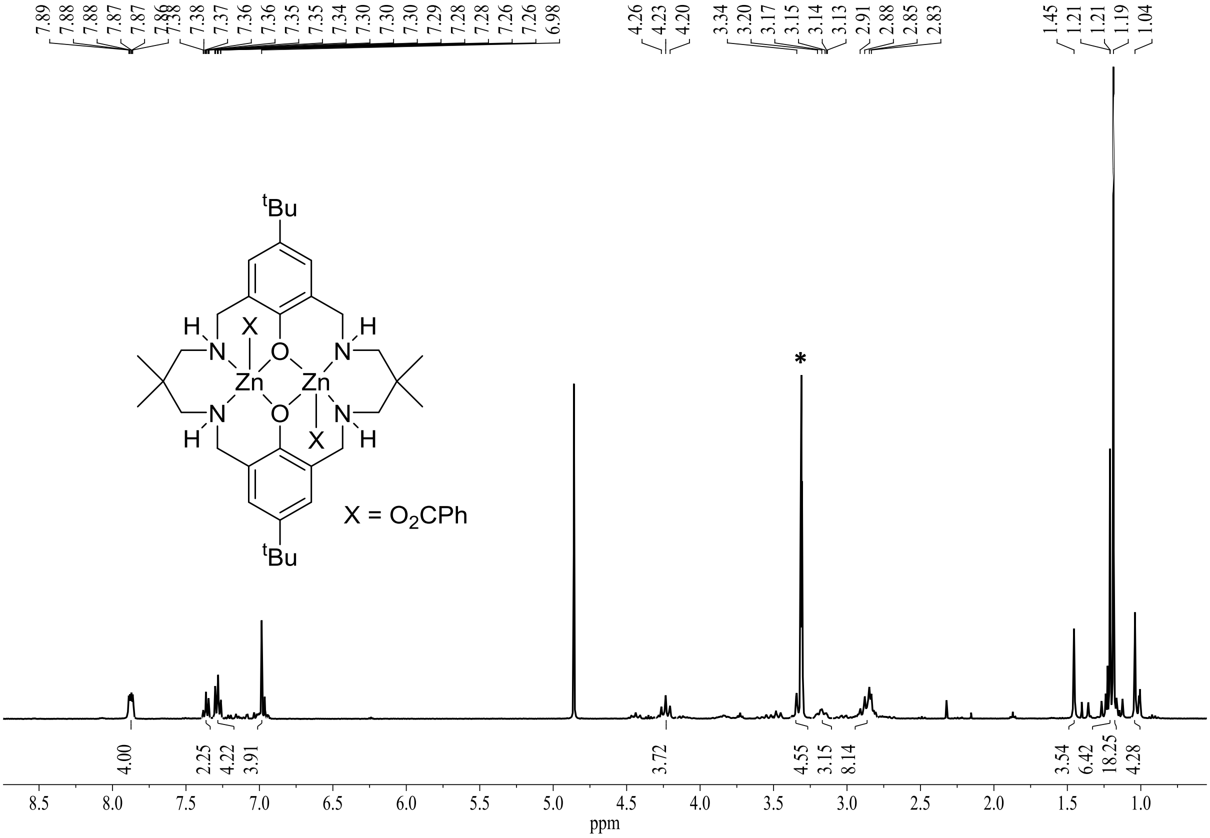

Figure S7. ^{1}H NMR of **3** in d$_4$–methanol solvent at 298 K. NMR spectra were collected at high dilution as **3** displays poor solubility in d$_4$–methanol. The minor peaks present are attributed to the presence of conformational isomers (data available at 10.14469/hpc/2151).

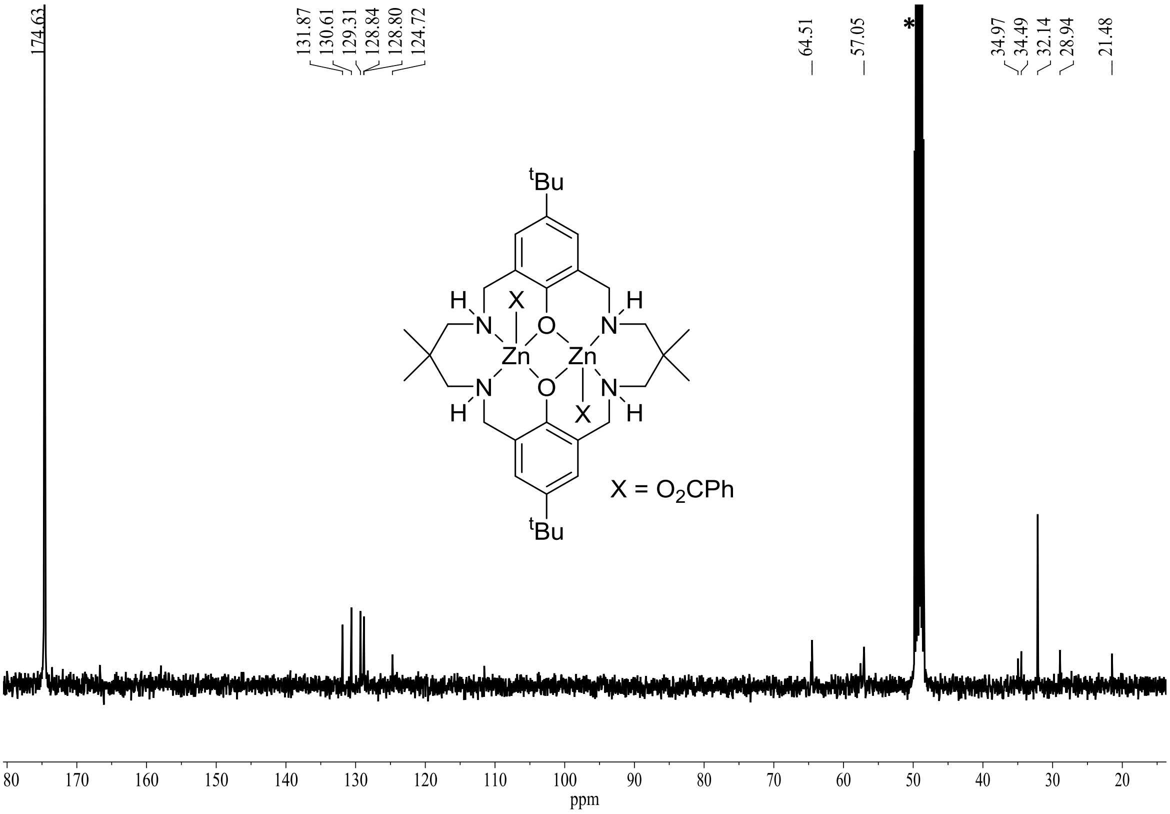

Figure S8. ^{13}C NMR of **3** in d$_4$–methanol solvent at 298 K (data available at 10.14469/hpc/2151).

DFT Calculations

Consideration of Potential Ligand Conformers

All calculations were performed using the Gaussian09 suite of codes. Calculations were carried out at the DFT level of theory, using hybrid functional ωB97XD. All atoms, including zinc, have been described with a 6-31G(d) basis set. Geometry optimisations were carried out without any symmetry restrictions and at the reaction temperature. Conductor-like polarisable continuum model (cpcm) was used with DCM as the solvent to model solvation in CHO.[1] The nature of the extrema was verified with analytical frequency calculations: the stationary points (minima) do not feature imaginary modes, and all transition states reveal precisely one imaginary mode corresponding to the intended reaction. For $\mathbf{V^{CO2}}$ and $\mathbf{IV^{CO2'}}$, IRC calculations were performed, which also confirmed the identity of the transition state.

Due to the high flexibility of the ligand in solution, different conformations of complex **1** were calculated considering both the “S” and “bowl” shapes (according to the X-ray structures obtained for this family of complexes). Optimisation of complex **1**, using a range of solvents (C_6H_6, DCM, THF) and temperatures (298.15, 333.15 and 373.15 K), found the “bowl” shape conformation to be favoured in all cases (by 2-6 kcal.mol^{-1}), versus the “S” shape. Although the “S” shape conformation was observed by X-ray diffraction studies for complex **1** (Figure S29), the presence of broad NMR spectra (Figure S1, S6) and a small energy difference between the two structures, calculated by DFT, suggests these two conformations are fluxional under the reaction conditions. A subsequent and detailed study of the key intermediates and transition states in the protonolysis reaction pathway was carried out for both the “S” and “bowl” shape conformations of complex **1** (ωb97xd/6-31G(d)/cpcm=DCM/Temp=353.15 K). These results confirmed that the “bowl” shaped conformation of the ligand allowed a significantly lower energy pathway for both the first and second protonolysis reactions.

The “bowl” shape conformation of the ligand was therefore subsequently used in all reaction profiles. In the case of CO_2 insertion, this conformation allows the approach of a CO_2 molecule at the convex face, leading to $\mathbf{II^{CO2}}$ and $\mathbf{II^{CO2'}}$ (Figure 3), which was not found possible with “S” shaped ligand geometries.

NBO Analysis of III-TS^{CO2}

NBO analysis of **III-TS^{CO2}** (Fig. S9) indicates an important orbital interaction (2nd order perturbation) between the Zn-C bonding orbital and the unfilled valence lone-pair orbital of the $C_{(CO2)}$ atom [BD(Zn-C) → LP*(C)$_{CO2}$ = 219 kcal/mol], suggesting the formation of a new C-C bond by nucleophilic attack of the Zn-$C_{(aryl)}$ bond to the $C_{(CO2)}$ atom. This is confirmed by a low Wiberg bond index of 0.25 for the Zn-$C_{(aryl)}$ bond (vs. 0.46 in **I**) and a high bond index, of 0.35, for the $C_{(aryl)}$-$C_{(CO2)}$ bond.

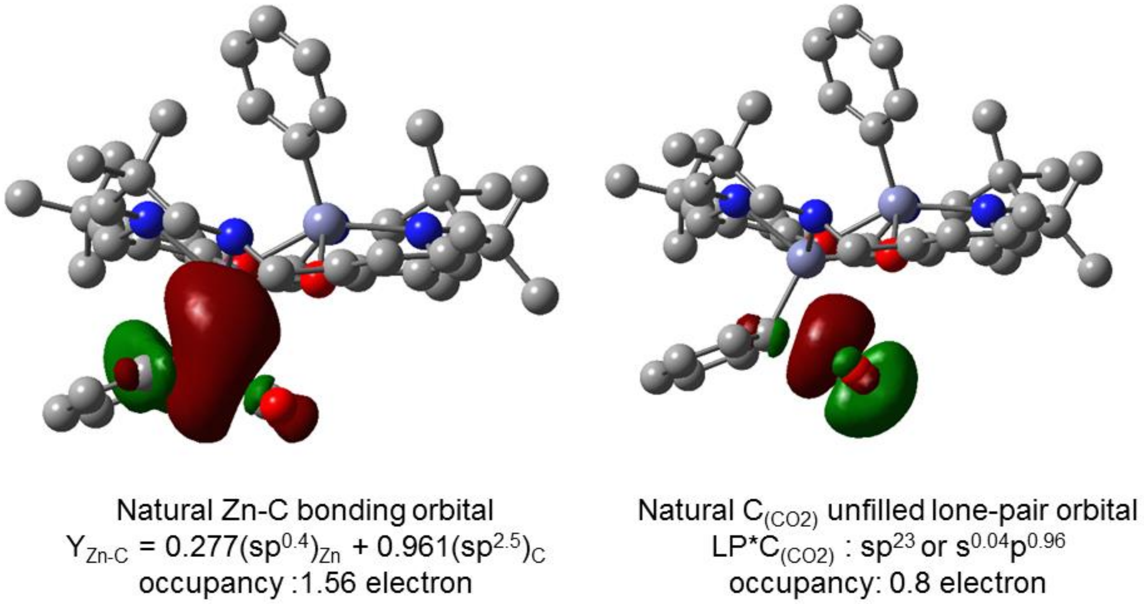

Figure S9. NBO analysis of **III-TS^{CO2}** (DFT protocol: ωb97xd/6-31G(d)/cpcm=toluene).

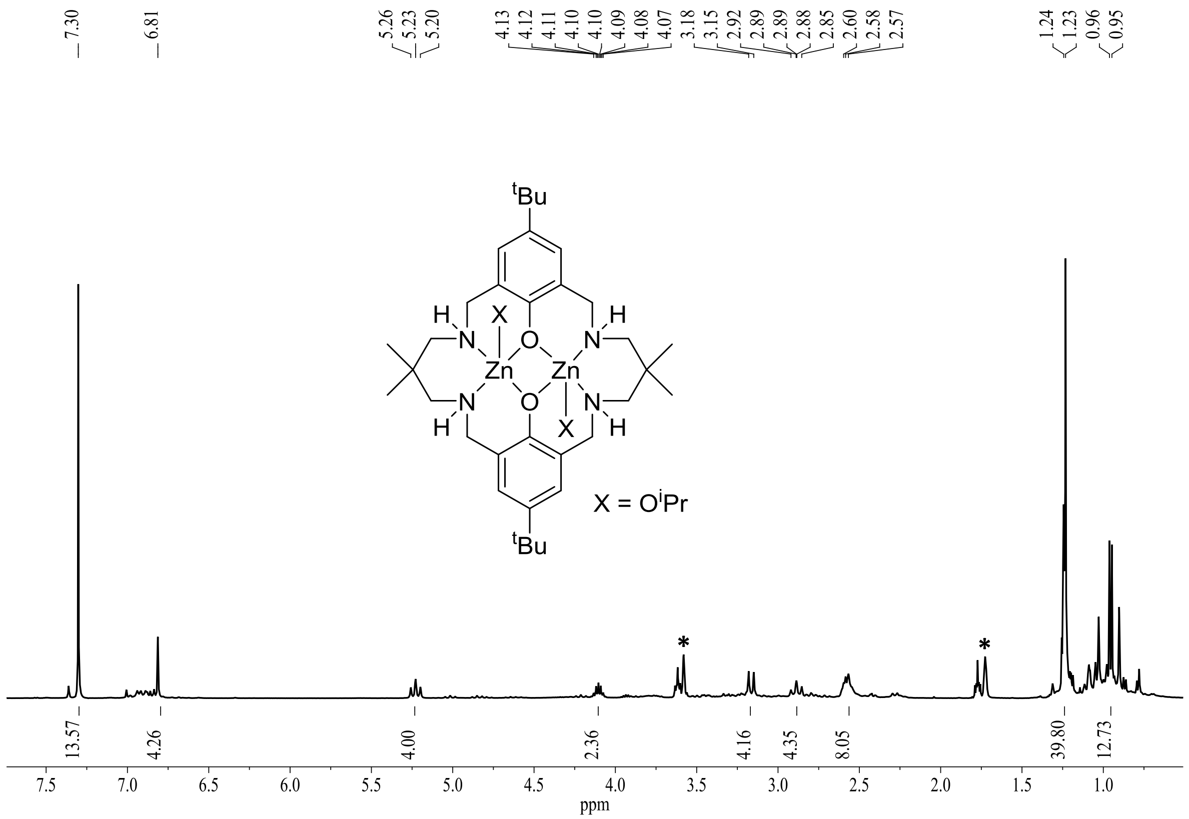

Figure S10. ^{1}H NMR of **4** in d_8-THF solvent at 298 K, prepared on an NMR scale from reaction of **1** with $^{i}PrOH$ (2 equiv.) (trace resonances of **1** are observed), (data available at 10.14469/hpc/2151).

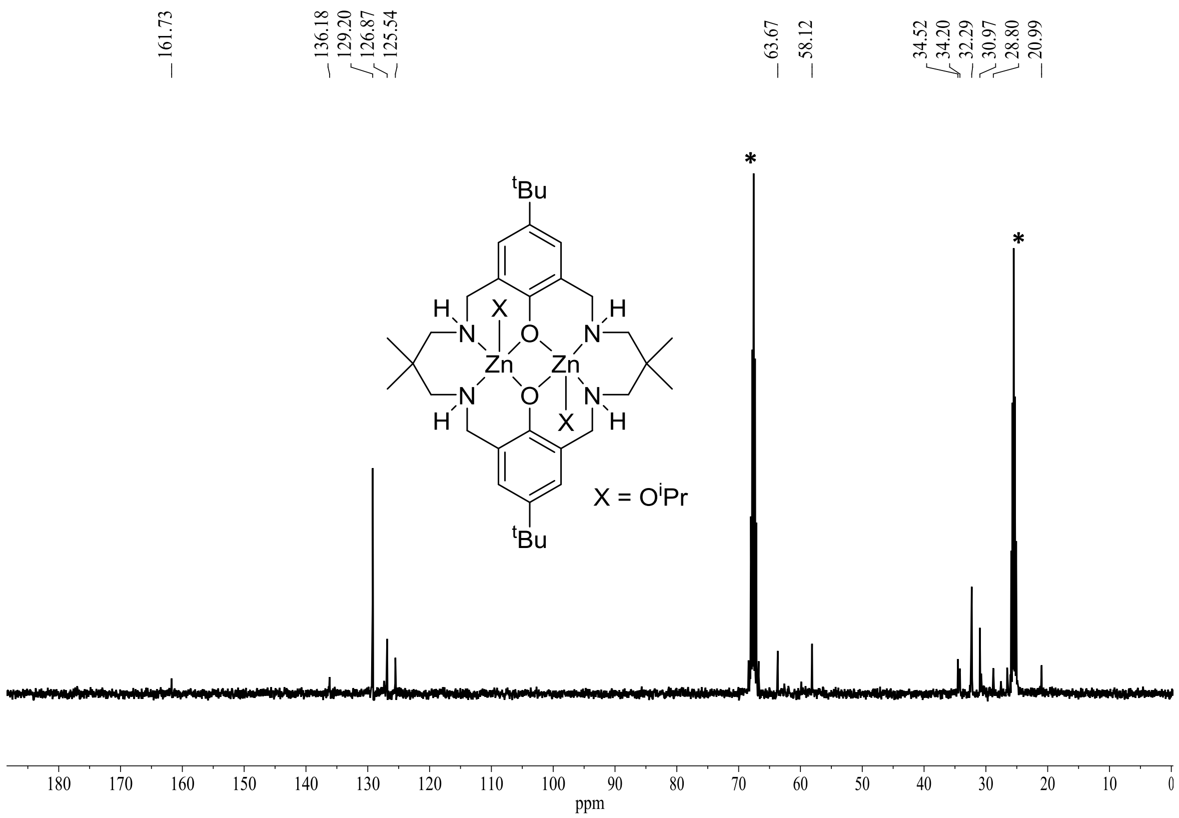

Figure S11. ^{13}C NMR of **4** in d_8-THF solvent at 298 K (data available at 10.14469/hpc/2151).

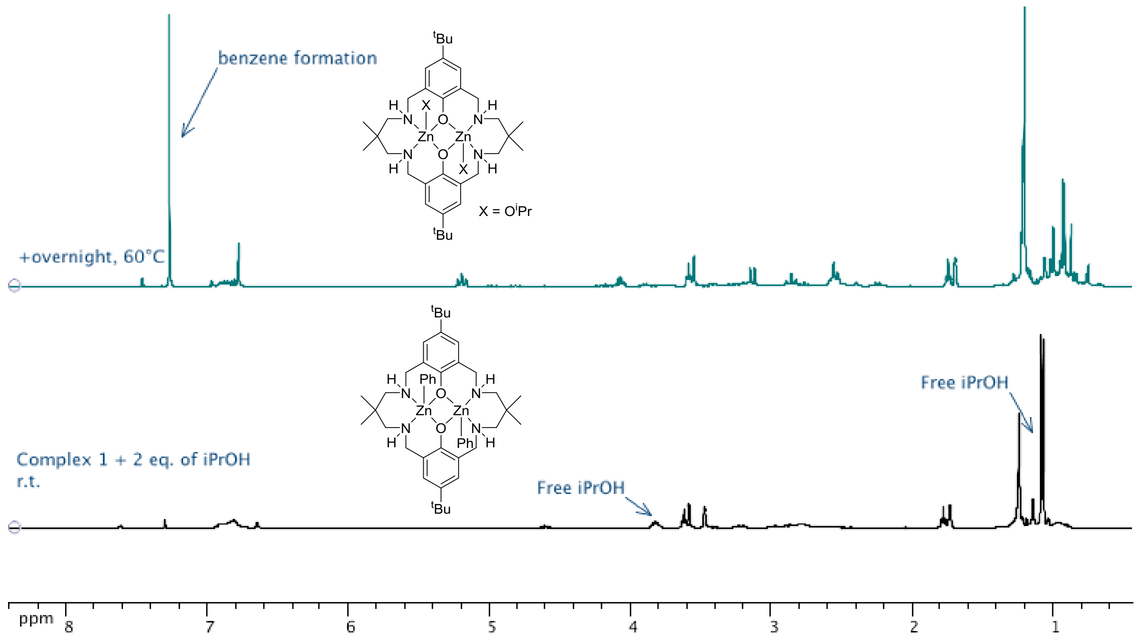

Figure S12. Overlay of ^{1}H NMR spectra for an NMR scale experiment, showing reaction of complex **1** in the presence of 2 equivalents of ^{i}PrOH in d$_8$-THF, overnight at 60 °C.

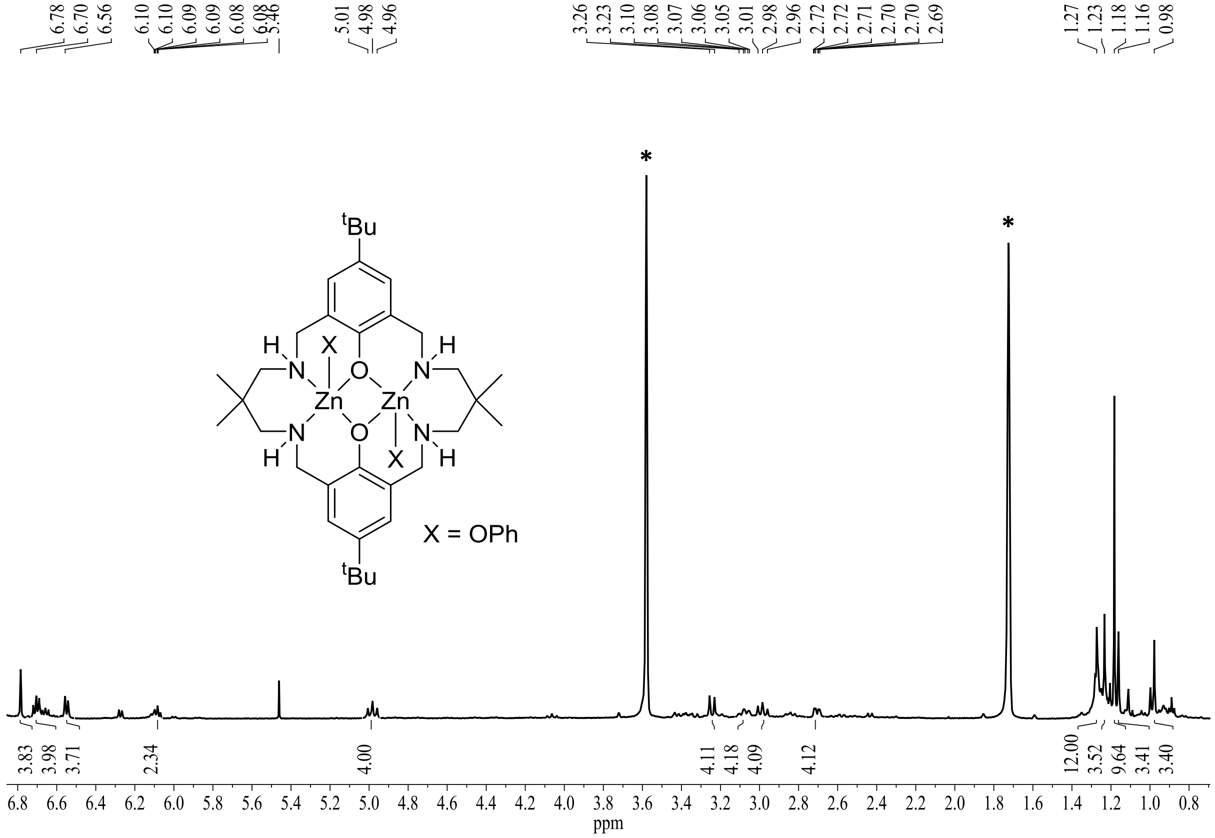

Figure S13. ^{1}H NMR of **5** in d$_8$-THF solvent at 328 K. Free phenol was observed due to the reaction of **5** with traces of water in the d$_8$-THF solvent (data available at 10.14469/hpc/2151).

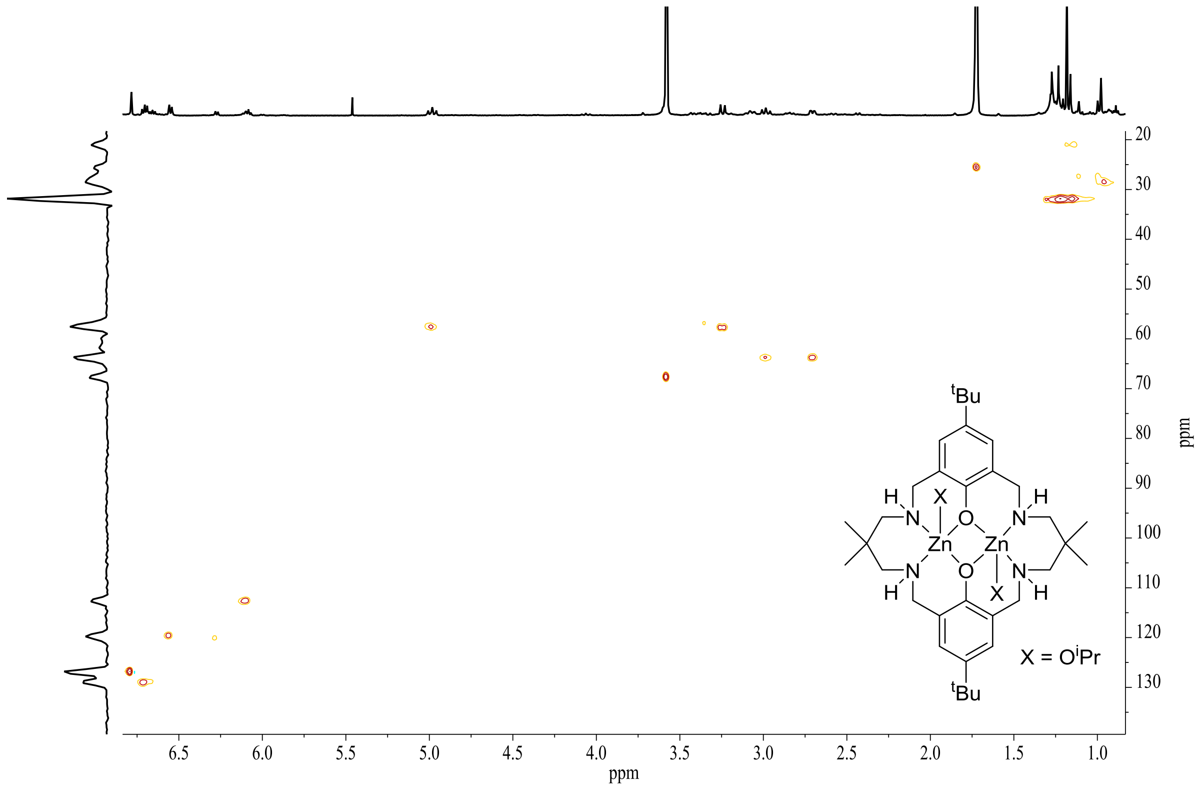

Figure S14. HSQC of **5** in d_8-THF solvent at 328 K. Due to the limited solubility of **5**, the ^{13}C NMR spectrum was too weak to identify all ^{13}C resonances.

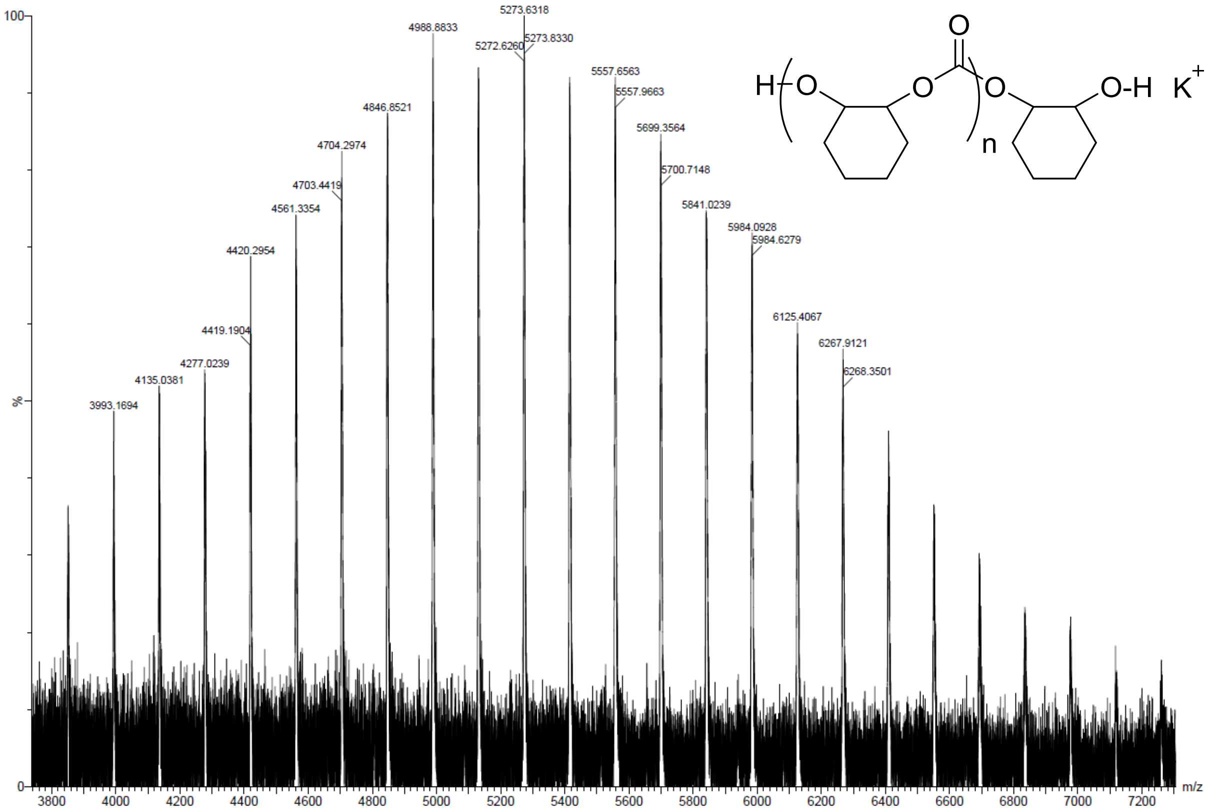

Figure S15. MALDI-ToF mass spectra of PCHC produced with complex **1**, showing formation of hydroxyl end-capped polymers [m/z = 116.1 (cyclohexanediol) + (142.15 x n)(CO_2-*alt*-CHO) + 39.0 (K^+)].

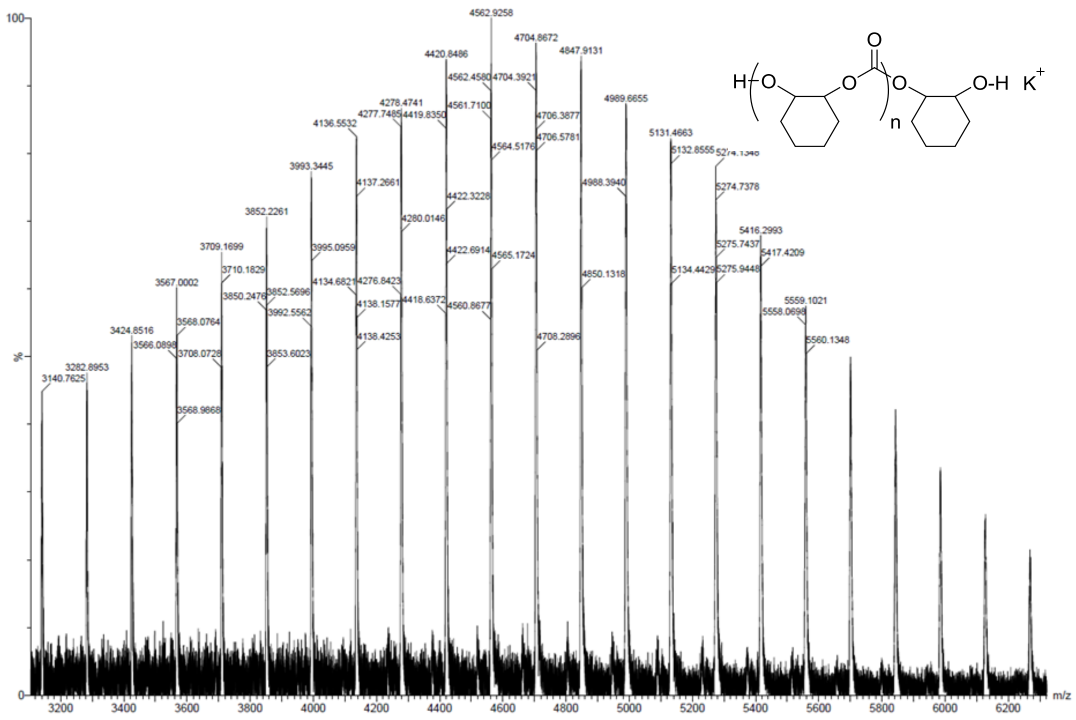

Figure S16. MALDI-ToF mass spectra of PCHC produced with complex **2**, showing formation of hydroxyl end-capped polymers [m/z = 116.1 (cyclohexanediol) + (142.15 x n)(CO_2-*alt*-CHO) + 39.0 (K^+)].

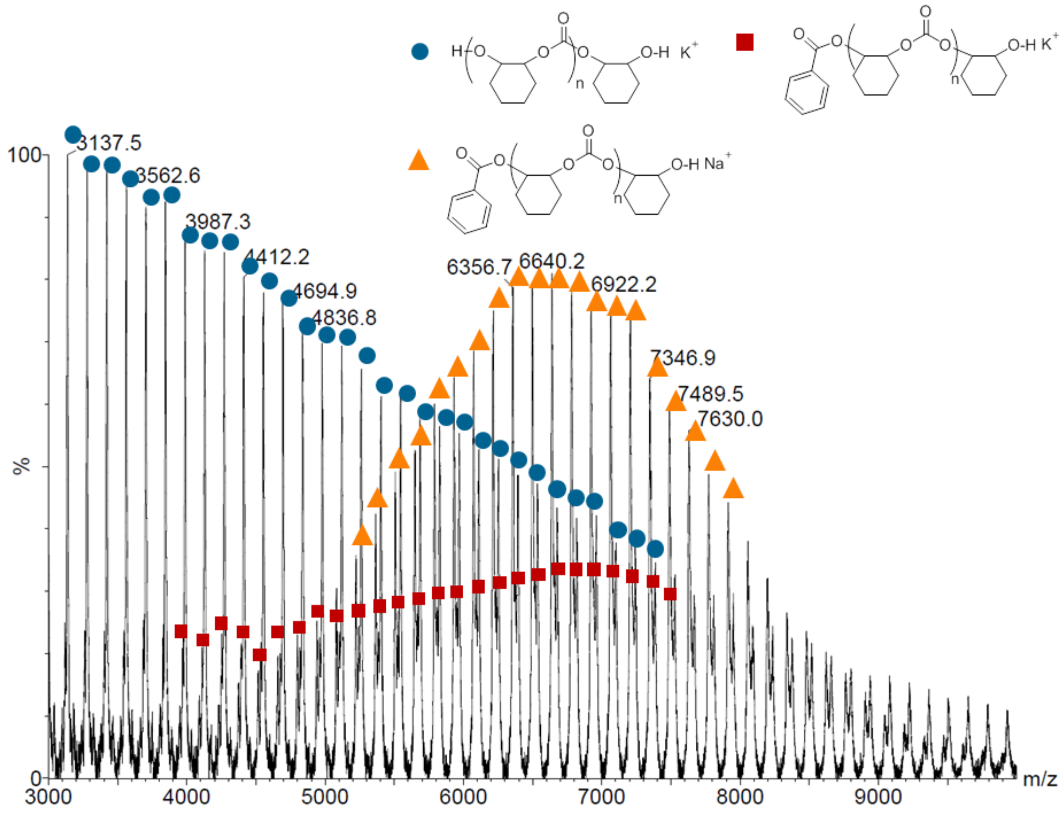

Figure S17. MALDI-ToF mass spectra of PCHC produced with complex **3**, showing formation of hydroxyl and benzoate end-capped polymers [m/z = 116.1 (cyclohexanediol) + (142.15 x n)(CO_2-*alt*-CHO) + 39.0 (K^+)].

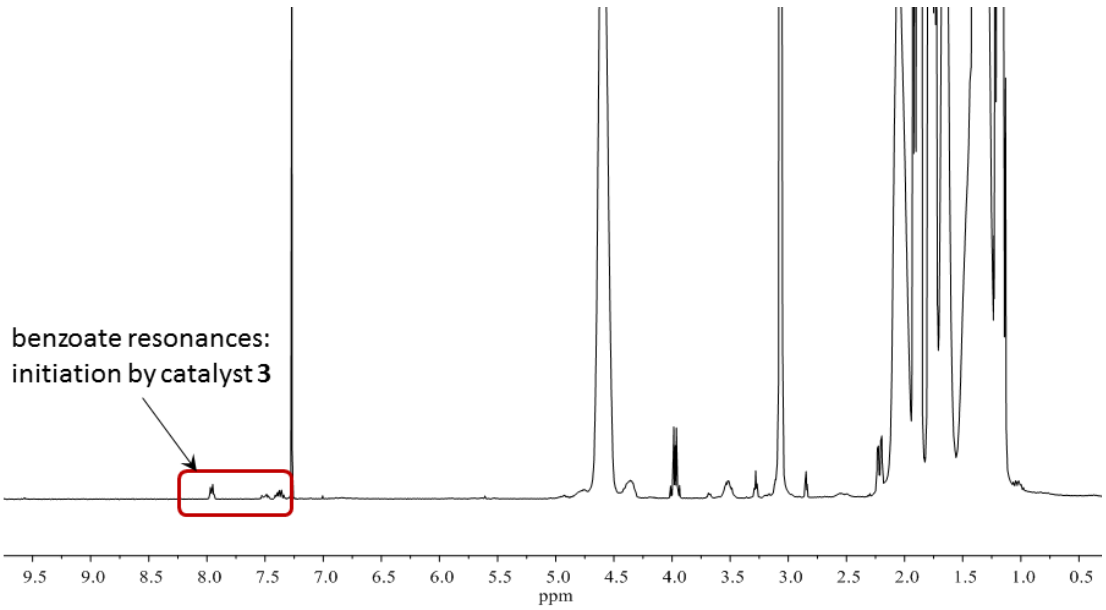

Figure S18. ^{1}H NMR spectrum of crude PCHC sample produced with **3**, in $CDCl_3$ at 298 K, showing the presence of aromatic resonances from the benzoate end group.

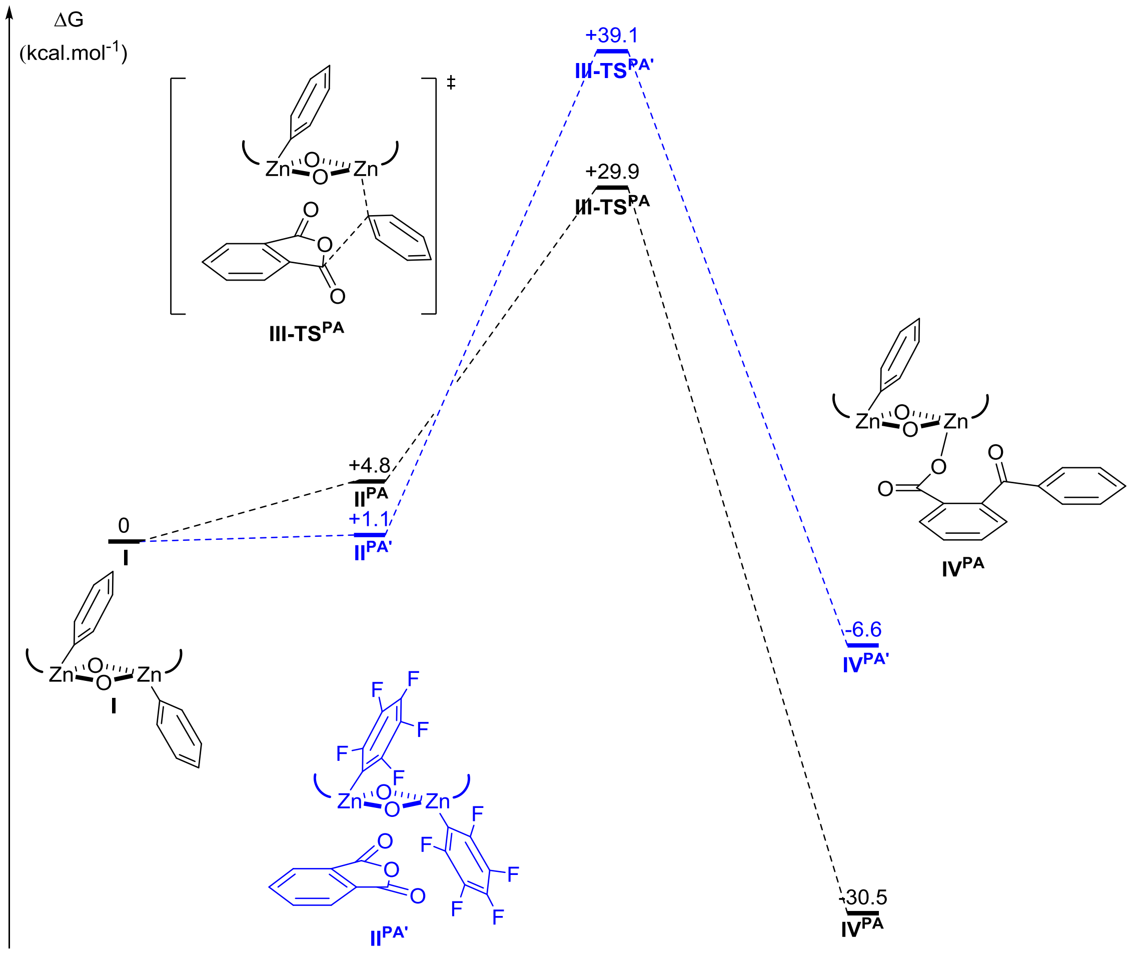

Figure S19. Potential energy surface for the first single ring opening of PA by the zinc aryl bond of **1**. (black) and **2** (blue); DFT protocol: wb97xd/6-31G(d)/cpcm=DCM/Temp=353.15 K (data available at 10.14469/hpc/2192 and10.14469/hpc/2198). The ancillary ligand structure is omitted for clarity.

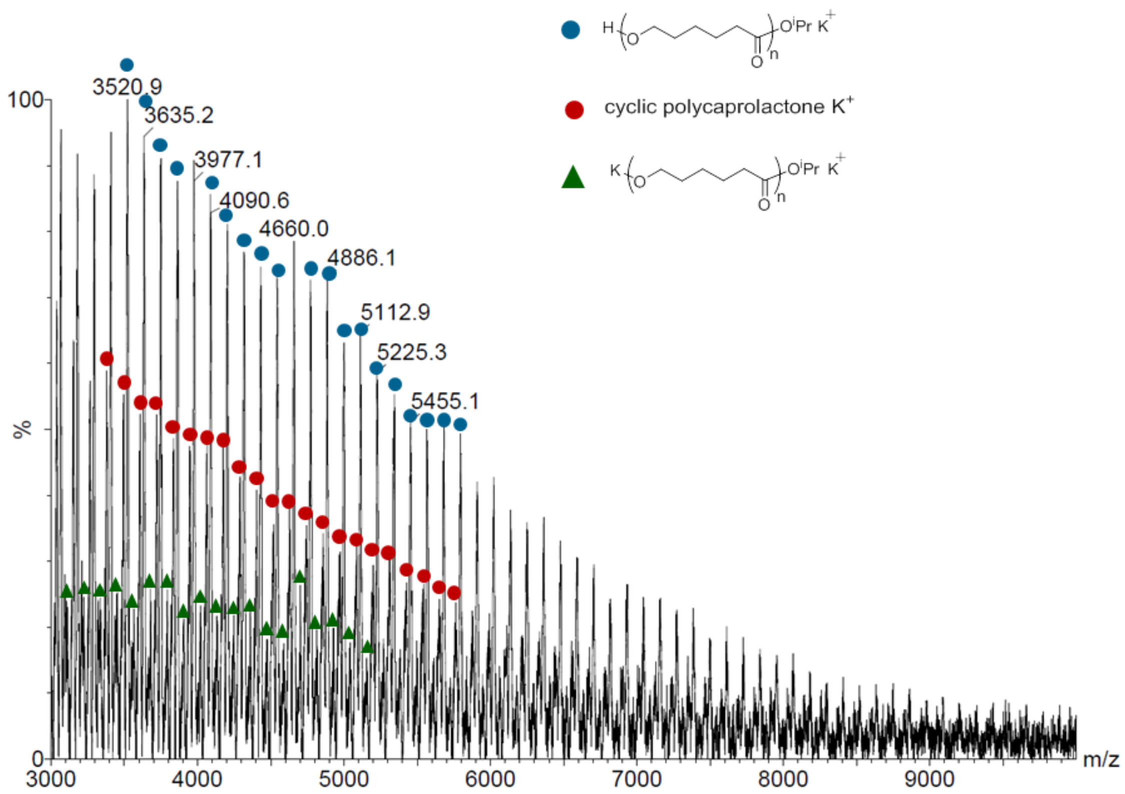

Figure S20. MALDI-ToF mass spectra of polycaprolactone produced with complex **1** at 80°C, showing formation of iso-propoxyl, hydroxyl end-capped polymers [m/z = 60.15 (iso-propanol) + (114.13 x n)(caprolactone) + 39.0 (K^+)].

Polymerisation Studies

CHO-CO_2 Copolymerisation Reactions

Cyclohexene oxide (2.5 mL, 25 mmol) and LZn_2Ph_2 (21 mg, 0.025 mmol) were added to a Schlenk tube. The vessel was evacuated and charged with 1 bar of CO_2 after which it was left stirring at 80 °C for 20h. The reaction mixture was quenched by exposure to air and a ^{1}H NMR spectrum of the crude reaction mixture was recorded in $CDCl_3$ solvent. Unreacted CHO was removed *in vacuo* and the product polycarbonate was then purified through dissolution in THF followed by precipitation from pentane, to produce a white powder.

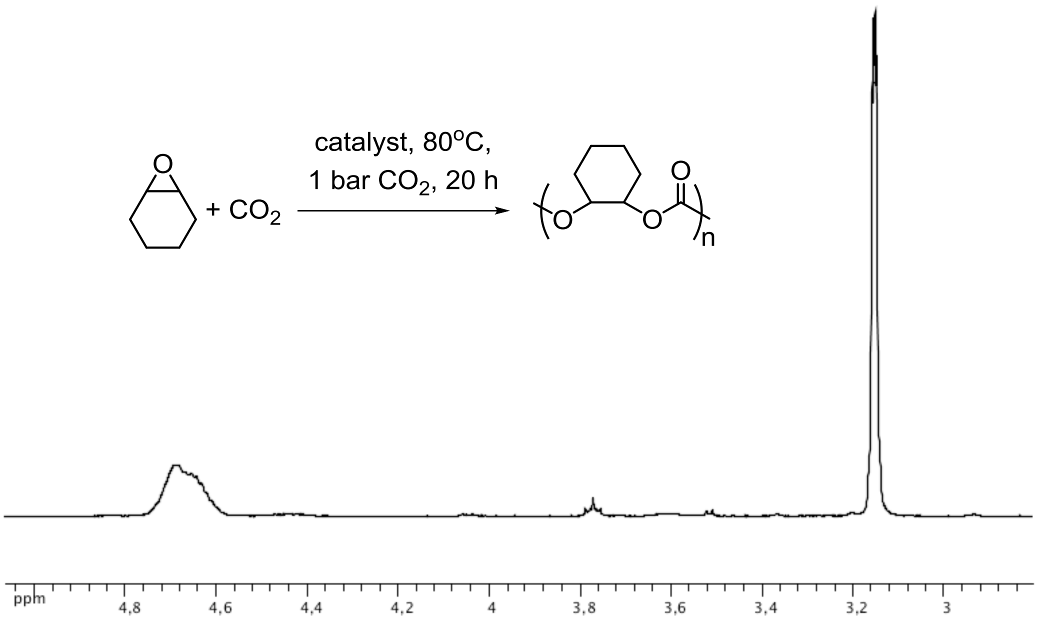

Figure S21. ^{1}H NMR spectrum of crude PCHC sample produced with **1**, in $CDCl_3$ at 298 K, showing formation of PCHC (4.68 ppm) with no ether linkages (3.4 ppm) and no formation of cyclic carbonate (4.1 ppm) by-products.

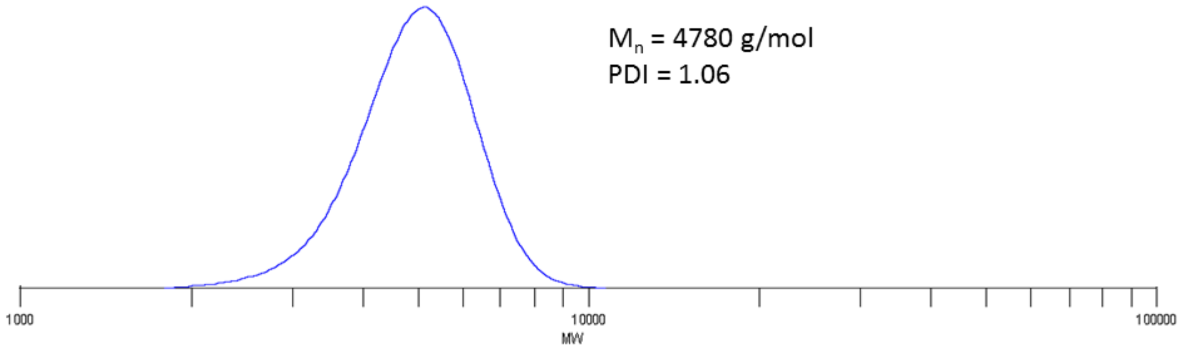

Figure S22. SEC plot of Mw for the polycarbonate formed by catalyst **1** (Table 1, Entry 1).

CHO-PA Copolymerisation Reactions

Under a nitrogen atmosphere in a glovebox, phthalic anhydride (177 mg, 1.20 mmol), cyclohexene oxide (1.1 mL, 10.8 mmol) and LZn_2Ph_2 (10 mg, 0.012 mmol) were added to a screw vial with Teflon cap. The vial was heated to 100 °C for 20 h, with constant stirring. A sample of the crude product was analysed by ^1H NMR spectroscopy in $CDCl_3$ solvent, to determine the conversion of the phthalic anhydride. The polymer was then purified by removal of excess CHO in vacuo, precipitation from pentane, followed by filtration to yield the polymer as an off-white powder. The crude sample shows complete consumption of the phthalic anhydride (7.97 and 7.87 ppm).

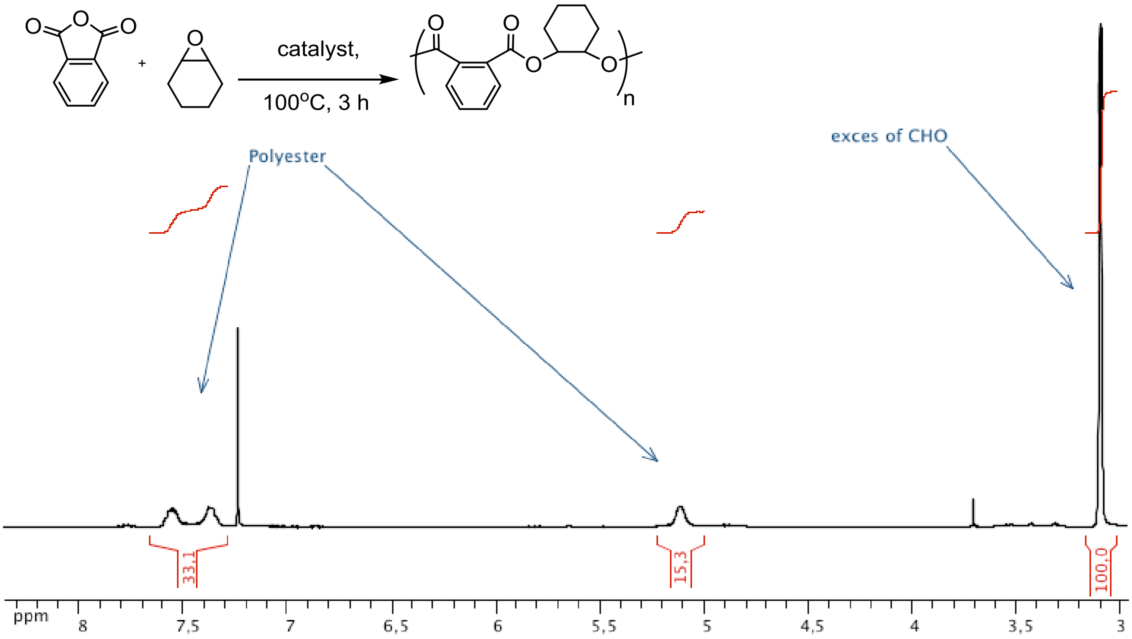

Figure S23. Expanded ^1H NMR spectrum of the crude CHO/PA copolymerisation reaction mixture in $CDCl_3$ at 298K, used to calculate the TON and TOF number achieved by the catalyst.

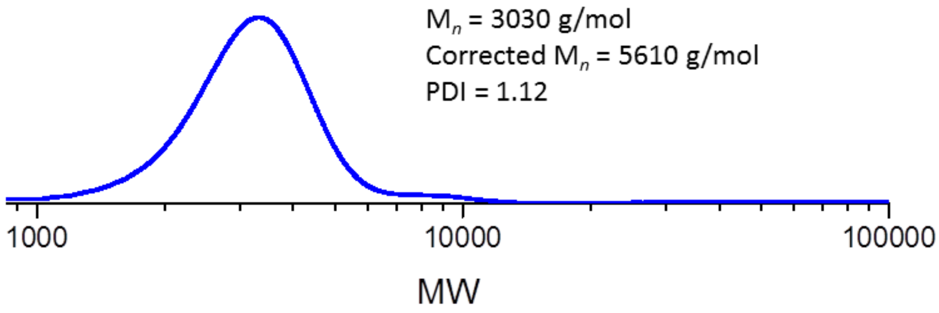

Figure S24. SEC plot of Mw for the polyester formed by catalyst **1** (Table 1, Entry 7).

ε-Caprolactone Polymerisation Reactions

Under a nitrogen atmosphere in a glovebox, ε-caprolactone (0.1 mL, 9.0 mmol), toluene (0.9 mL), LZn_2Ph_2 (15 mg, 0.018 mmol) and *iso*-propanol (5.5 μL, 0.072 mmol) were added to a screw vial. The vessel was heated at 80 °C for 10 minutes with constant stirring. The polymer product was purified by dissolution in THF, then precipitation from methanol to afford a white powder.

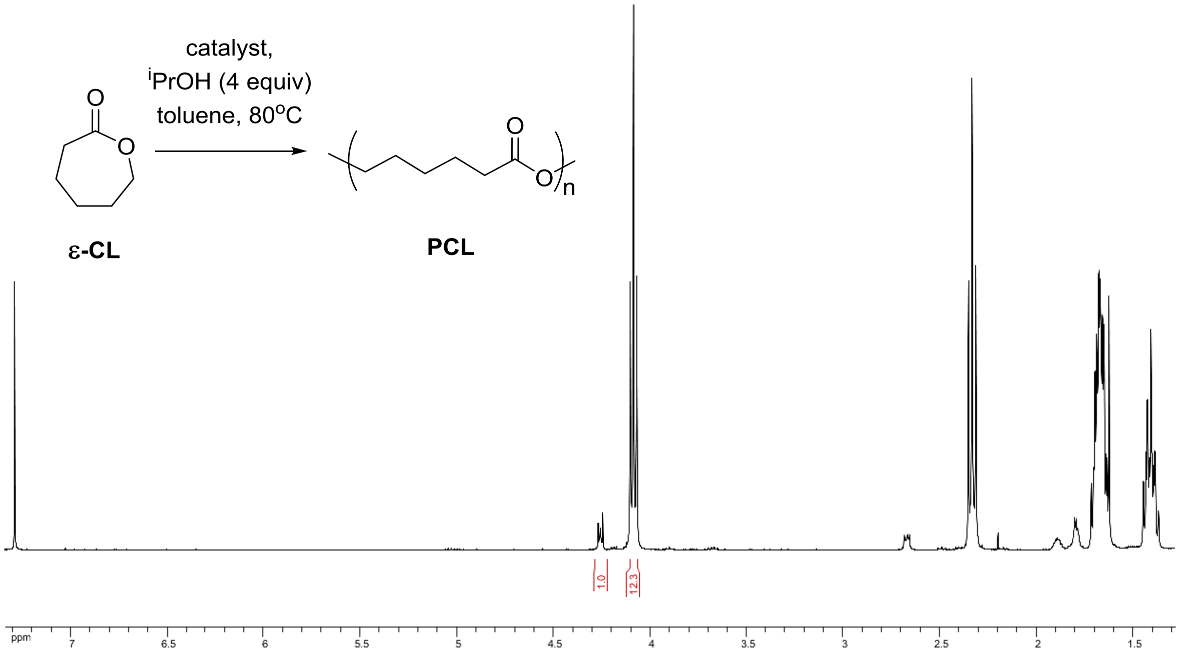

Figure S25. Expanded ^{1}H NMR spectrum of the crude ε-caprolactone polymerisation reaction mixture in $CDCl_3$ at 298K, showing the polycaprolactone (4.08 ppm) and ε-caprolactone (4.26 ppm) signals used to calculate the TON and TOF number achieved by the catalyst.

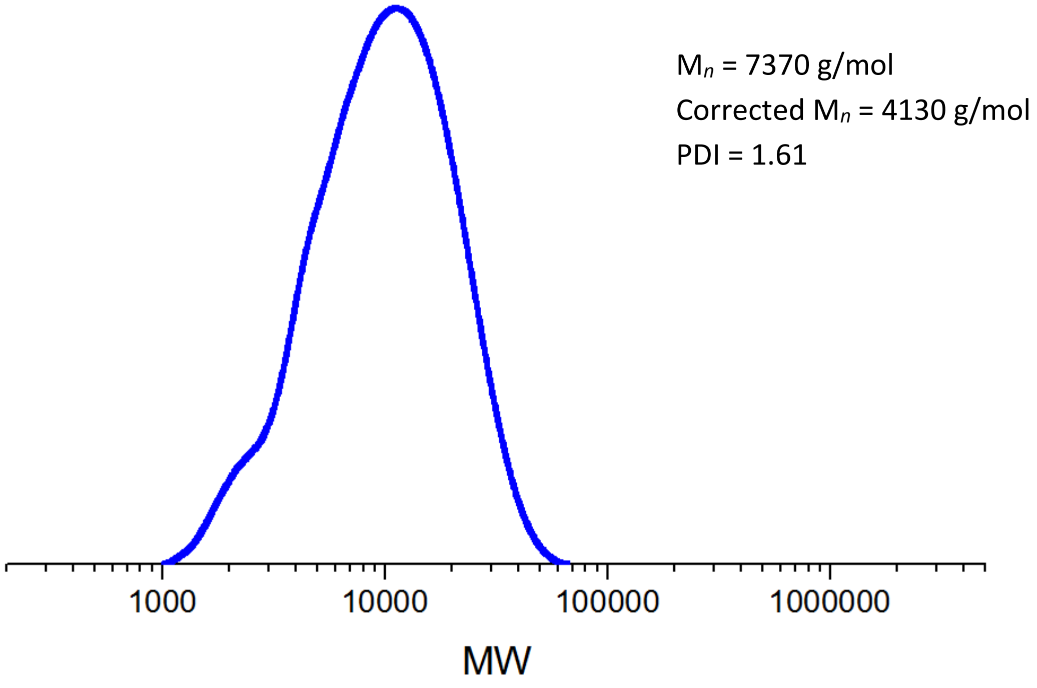

Figure S26. SEC plot of Mw for the polycaprolactone formed by catalyst **1** (Table 1, Entry 10).

rac-Lactide Polymerisation Reactions

Under a nitrogen atmosphere in the glovebox, LZn_2Ph_2 (10 mg, 0.012 mmol) was added to a screw capped vial, followed by a 1M solution of rac-lactide in THF (345 mg, 2.4 mmol in 2.39 mL), and *iso*-propanol (1.84 µL, 0.024 mmol), to bring the final concentration of rac-lactide in THF to 1M. The polymers were purified through recrystallisation from THF/methanol.

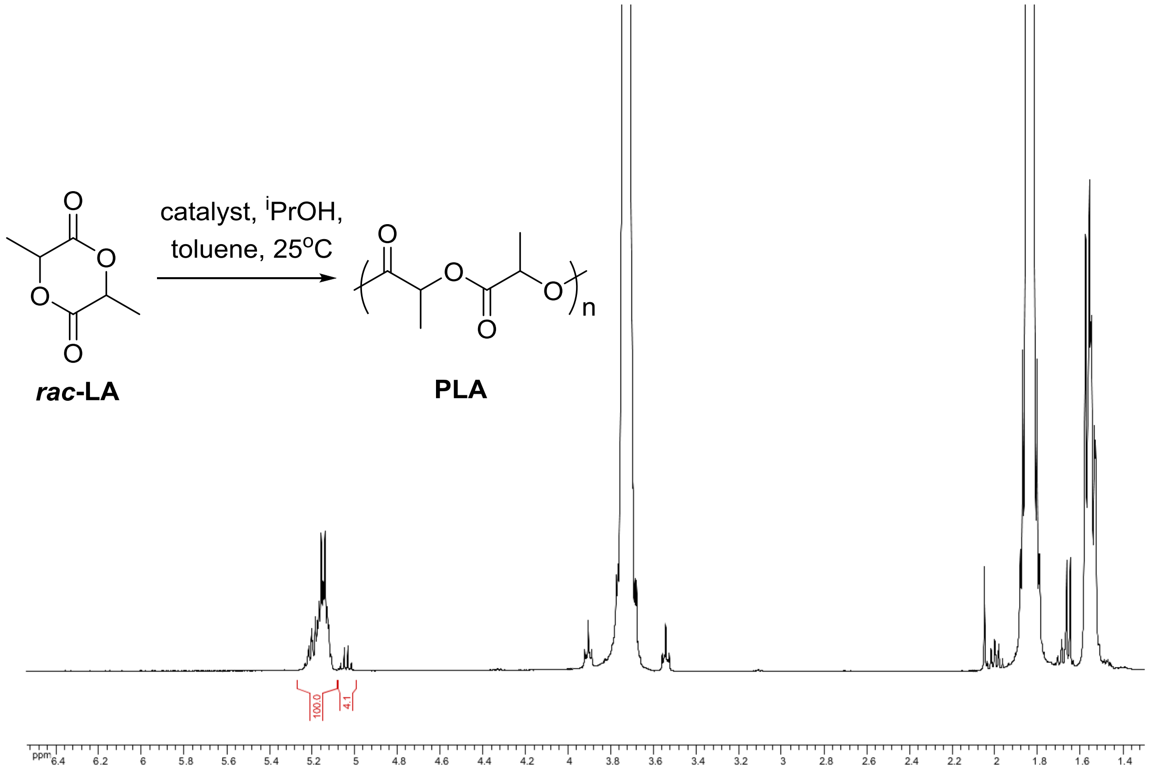

Figure S27. Expanded ^{1}H NMR spectrum of the crude rac-lactide polymerization reaction mixture in $CDCl_3$ at 298K, showing the polylactide (5.15 ppm) and rac-lactide (5.04 ppm) signals used to calculate the TON and TOF number achieved by the catalyst.

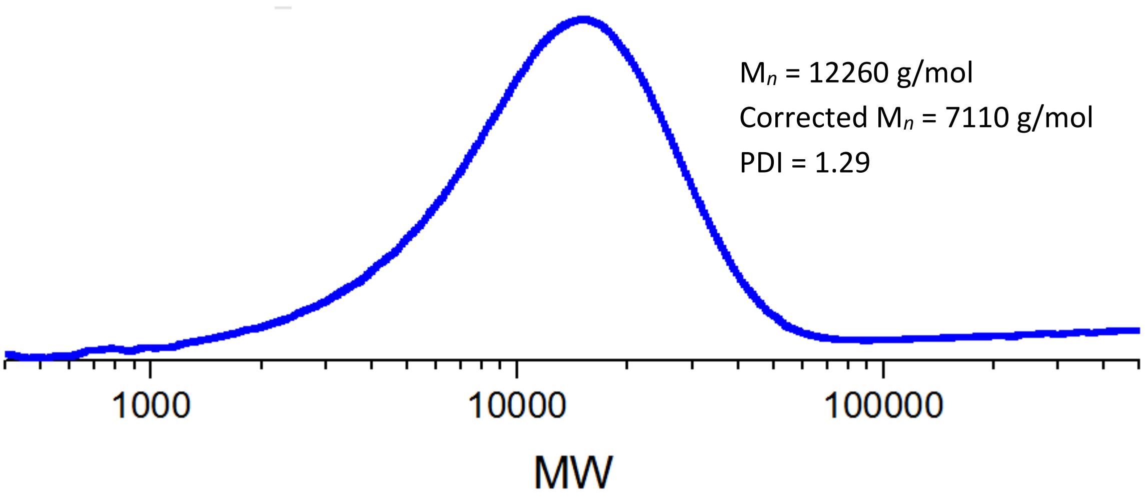

Figure S28. SEC plot of Mw for the polylactide formed by catalyst **1** (Table 1, entry 12).

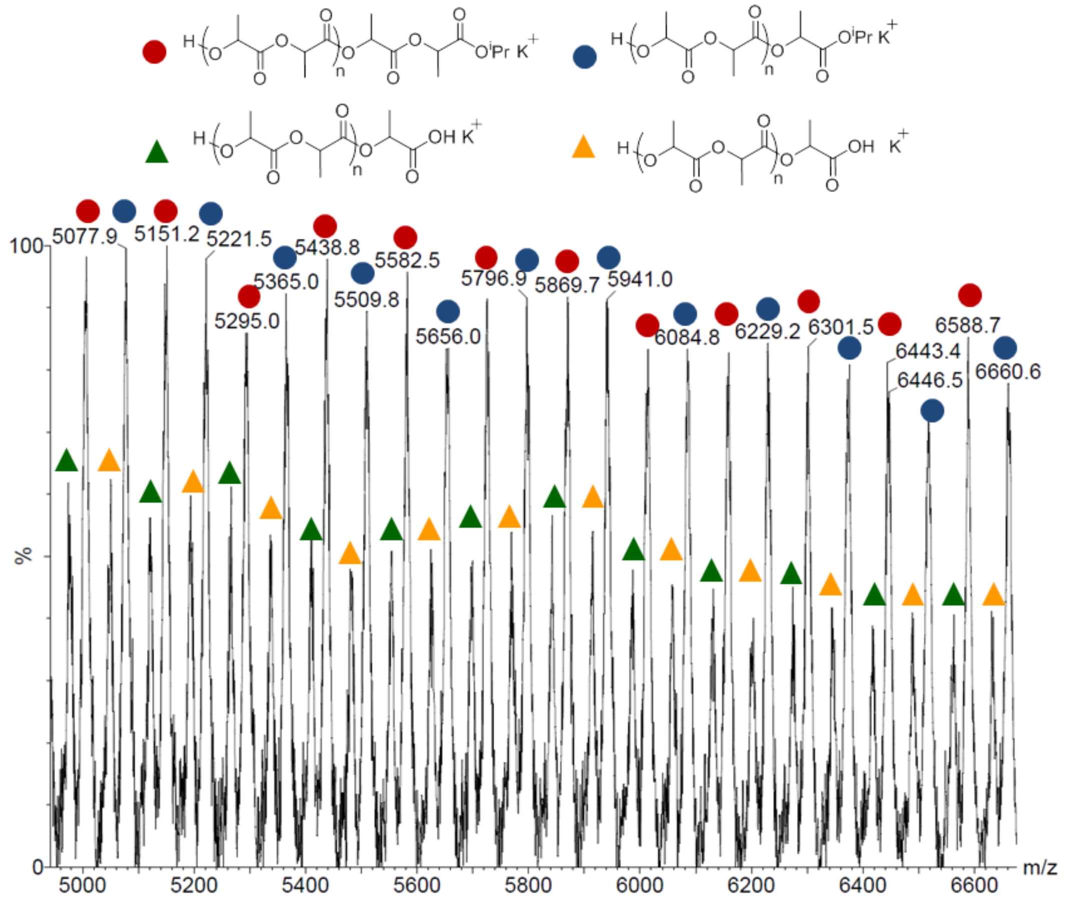

Figure S29. MALDI-ToF mass spectra of polylactide produced with complex **1** at 80°C, showing formation of iso-propoxyl and hydroxyl end-capped polymers [m/z = 60.15 (iso-propanol) + (144.1 x n)(lactide) + 39.0 (K^+)].

X-Ray Crystallography Data

Table S1 Comparative bond length data for complexes **1**, **2**, **3** and **5**.

Complex	Bond Lengths (Å)				
	**Zn-O**	**ArC-O**	**Zn-N**	**Zn-ligand[a]**	**Zn···Zn**
**1**	2.038 (1)<br>2.1707(9)	1.341(2)	2.162(1)<br>2.212(1)	2.016(1)	3.2745(3)
**2**	2.0385(9)<br>2.1291(9)	1.338(2)	2.129(1)<br>2.168(1)	2.049(1)	3.2066(2)
**3[b]**	2.007(2)<br>2.040(2)	1.351(3)	2.142(2)<br>2.153(2)	1.962(2)	3.1099(5)
**5**	2.0445(9)<br>2.0537(9)	1.348(1)	2.109 (1)<br>2.120 (1)	1.9545(9)	3.1824(2)

a) Where ligand refers to Ph in **1**, C_6F_5 in **2**, O_2CPh in **3** and OPh in **4**; b) Values given are for Zn(1).

Table S2 Comparative bond angle data for complexes **1**, **2**, **3** and **5**.

Complex	Bond Angles (°)					
	**O-Zn-O**	**Zn-O-Zn**	**ArO-Zn-ligand[a]**	**N-Zn-ligand[a]**	**ArO-Zn-N**	**N-Zn-N**
**1**	77.90(4)	102.10(4)	106.63(5)<br>119.73(5)	107.14(5)<br>110.85(5)	81.65(4)<br>82.82(4)<br>132.61(4)<br>142.44(4)	88.45(4)
**2**	79.42(4)	100.58(4)	105.31(5)<br>110.06(5)	107.74(5)<br>114.80(5)	82.37(4)<br>83.03(4)<br>134.69(4)<br>146.08(4)	89.91(4)
**3[b]**	80.37(7)	100.16(8)	108.93(8)<br>113.14(8)	94.62(9)<br>96.75(9)	88.20(9)<br>90.51(8)<br>152.09(9)<br>154.30(8)	88.94(9)
**5**	78.11(4)	101.89(4)	110.35(4)<br>112.80(4)	98.83(4)<br>101.91(4)	85.74(4)<br>86.08(4)<br>147.46(4)<br>147.89(4)	93.30(4)

a) Where ligand refers to Ph in **1**, C_6F_5 in **2**, O_2CPh in **3** and OPh in **4**; b) Values given are for Zn(1).

The X-ray crystal structure of 1

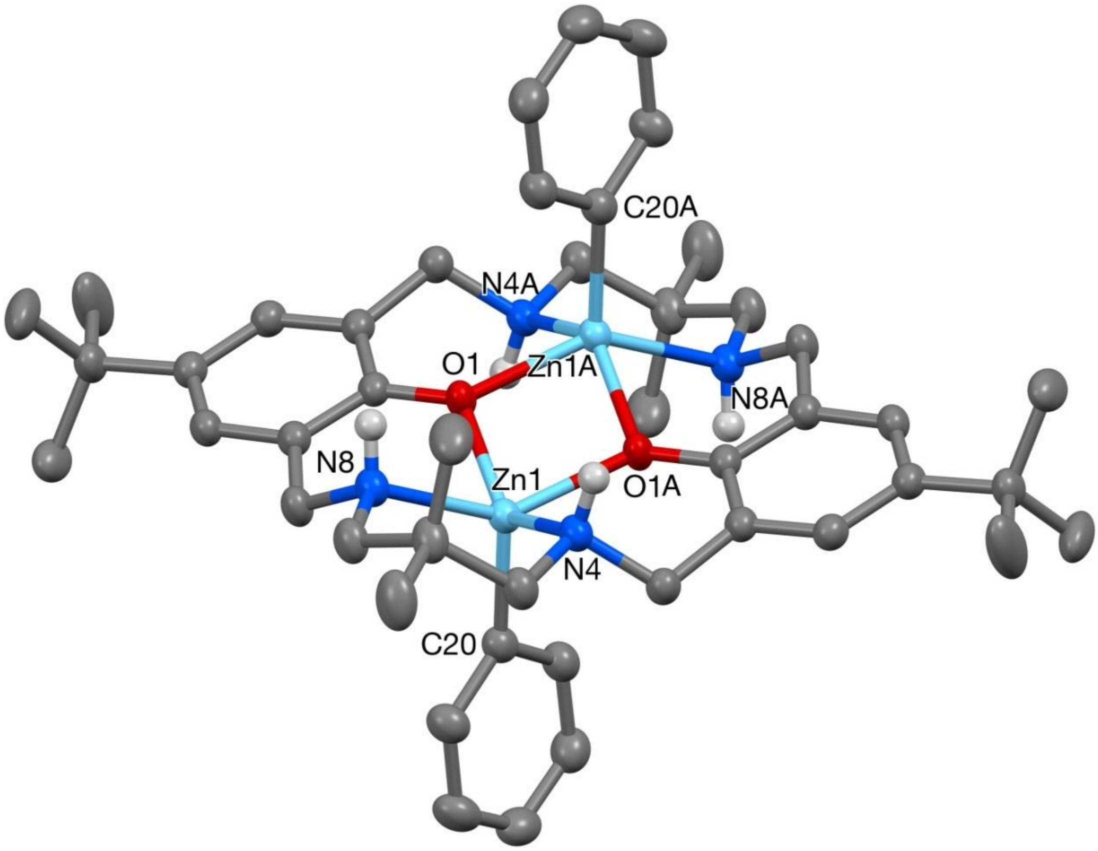

Figure S30. The structure of the C_i-symmetric complex present in the crystals of **1** (50% probability ellipsoids).

The complex and the included benzene solvent molecule in the structure of **1** were found to be situated across independent centres of symmetry. The C16-based *t*-butyl group was found to be disordered. Two orientations were identified of *ca*. 85 and 15% occupancy, their geometries were optimised, the thermal parameters of adjacent atoms were restrained to be similar, and only the non-hydrogen atoms of the major occupancy orientation were refined anisotropically (those of the minor occupancy orientation were refined isotropically). The two unique N–H hydrogen atoms were located from ΔF maps and refined freely subject to an N–H distance constraint of 0.90 Å.

Crystal data for **1**: $C_{46}H_{64}N_4O_2Zn_2 \cdot C_6H_6$, M = 913.86, monoclinic, $P2_1/n$ (no. 14), a = 11.42233(8), b = 12.79857(8), c = 16.88310(13) Å, β = 108.8415(8)°, V = 2335.88(3) Å^3, Z = 2 [C_i symmetry], D_c = 1.299 g cm^{-3}, μ(Cu-Kα) = 1.593 mm^{-1}, T = 173 K, colourless blocks, Oxford Diffraction Xcalibur PX Ultra diffractometer; 4582 independent measured reflections (R_{int} = 0.0201), F^2 refinement,[2] R_1(obs) = 0.0288, wR_2(all) = 0.0823, 4343 independent observed absorption-corrected reflections [$|F_o| > 4\sigma(|F_o|)$, $2\theta_{max}$ = 145°], 297 parameters. CCDC 1498754.

The X-ray crystal structure of 2

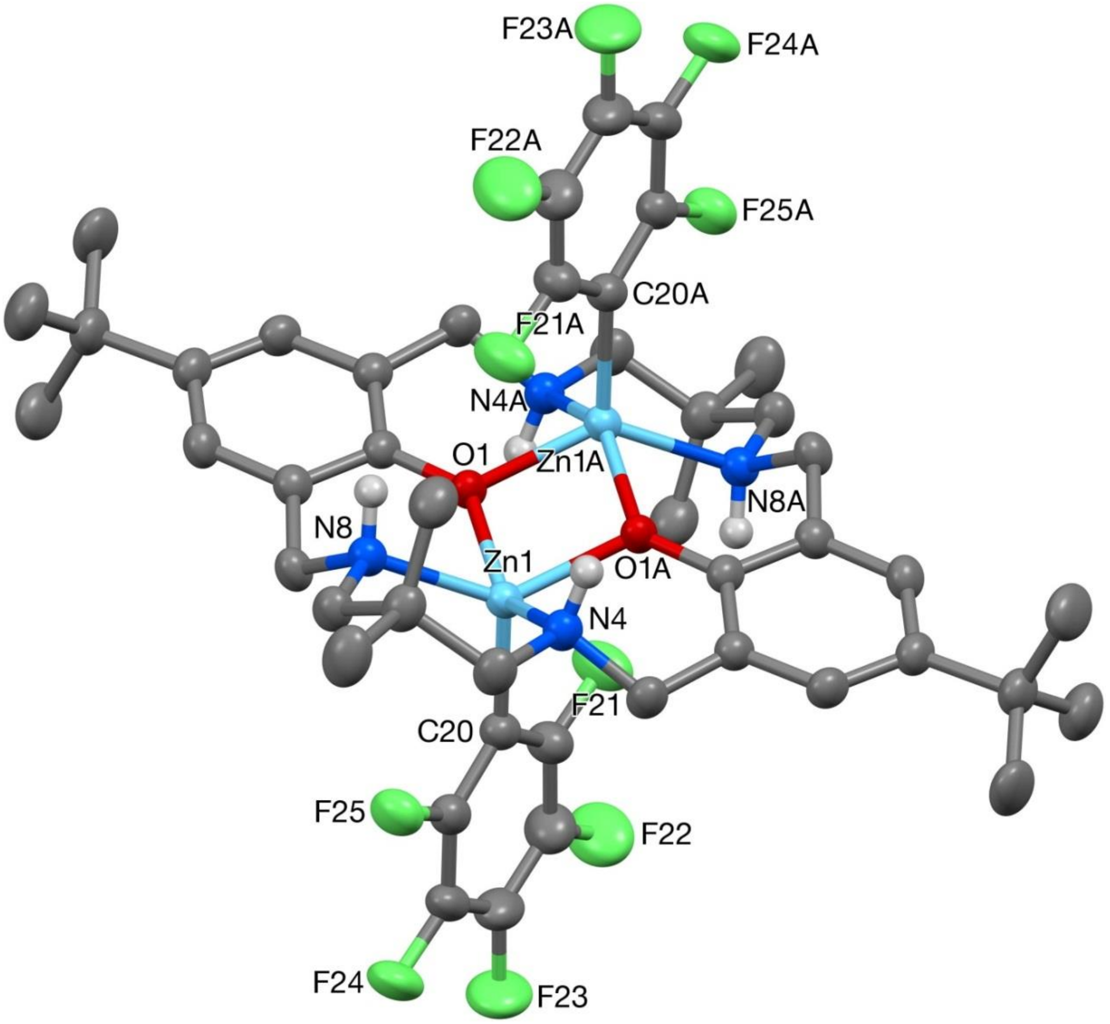

Figure S31. The structure of the C_i-symmetric complex present in the crystals of **2** (50% probability ellipsoids).

The complex in the structure of **2** was found to be situated across a centre of symmetry. Two of the three unique included benzene solvent molecules were found to be disordered, and in each case two orientations were identified of *ca*. 71:29 and 64:36% occupancy for the C41- and C51-based molecules respectively. The geometries of all four orientations were optimised, the thermal parameters of adjacent atoms were restrained to be similar, and only the non-hydrogen atoms of the major occupancy orientations were refined anisotropically (those of the minor occupancy orientations were refined isotropically). The two unique N–H hydrogen atoms were located from Δ*F* maps and refined freely subject to an N–H distance constraint of 0.90 Å.

Crystal data for **2**: $C_{46}H_{54}F_{10}N_4O_2Zn_2 \cdot 6(C_6H_6)$, M = 1484.32, monoclinic, $P2_1/n$ (no. 14), a = 12.66978(6), b = 23.84792(12), c = 12.82336(6) Å, β = 105.5651(5)°, V = 3732.46(3) Å^3, Z = 2 [C_i symmetry], D_c = 1.321 g cm^{-3}, μ(Cu-Kα) = 1.409 mm^{-1}, T = 173 K, colourless blocks, Oxford Diffraction Xcalibur PX Ultra diffractometer; 7316 independent measured reflections (R_{int} = 0.0182), F^2 refinement,[X1] R_1(obs) = 0.0303, wR_2(all) = 0.0847, 6693 independent observed absorption-corrected reflections [$|F_o| > 4\sigma(|F_o|)$, $2\theta_{max}$ = 145°], 461 parameters. CCDC 1498755.

The X-ray crystal structure of 3

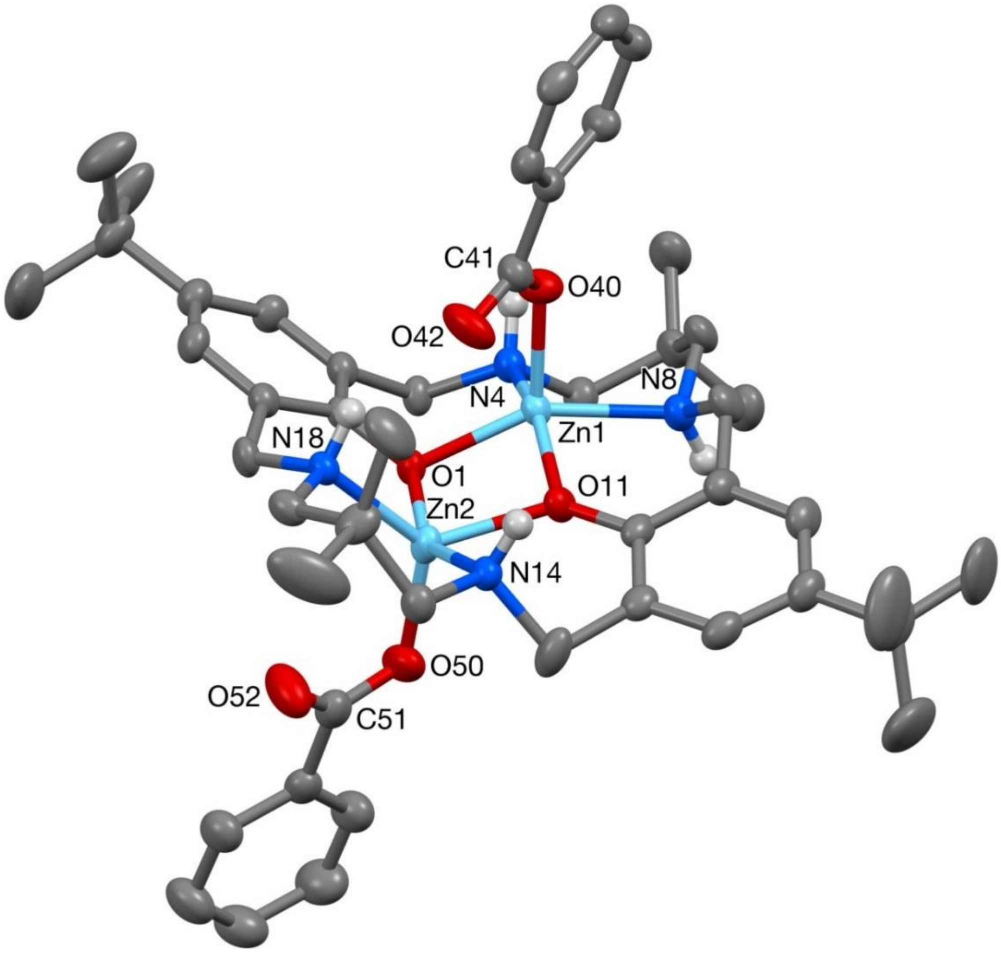

Figure S32. The crystal structure of **3** (50% probability ellipsoids).

The N14–C15 portion of the macrocyclic ring in the structure of **3** was found to be disordered. Two orientations were identified of *ca*. 58 and 42% occupancy, their geometries were optimised, the thermal parameters of adjacent atoms were restrained to be similar, and only the non-hydrogen atoms of the major occupancy orientation were refined anisotropically (those of the minor occupancy orientation were refined isotropically). The C33-based *t*-butyl moiety was found to be disordered. Two orientations were identified for the methyl groups of *ca*. 92 and 8% occupancy, their geometries were optimised, the thermal parameters of adjacent atoms were restrained to be similar, and only the non-hydrogen atoms of the major occupancy orientation were refined anisotropically (those of the minor occupancy orientation were refined isotropically). The C61-based included benzene solvent molecule was found to be disordered across a centre of symmetry, and two unique orientations of *ca*. 30 and 20% occupancy were identified (with the action of the inversion centre generating two further orientations of the same occupancies). The geometries of both orientations were optimised, the thermal parameters of adjacent atoms were restrained to be similar, and all of the atoms were refined isotropically. The N–H hydrogen atoms of the three ordered N–H units (based on N4, N8, and N18) were located from ΔF maps and refined freely subject to an N–H distance constraint of 0.90 Å, whilst for the disordered N14-based unit the N–H hydrogen for each of the partial occupancy orientations was added in an idealised position and allowed to ride on its parent atom at an N–H distance of 0.90 Å.

Crystal data for **3**: $C_{48}H_{64}N_4O_6Zn_2\cdot{}^1/_2(C_6H_6)$, M = 962.82, monoclinic, $P2_1/c$ (no. 14), a = 12.2452(3), b = 25.0464(6), c = 15.9078(4) Å, β = 95.819(2)°, V = 4853.75(19) Å^3, Z = 4, D_c = 1.318 g cm^{-3}, μ(Mo-Kα) = 1.040 mm^{-1}, T = 173 K, colourless blocks, Agilent Xcalibur 3 E diffractometer; 9738 independent measured reflections (R_{int} = 0.0225), F^2 refinement,[X1] R_1(obs) = 0.0429, wR_2(all) = 0.1111, 7657 independent observed absorption-corrected reflections [$|F_o| > 4\sigma(|F_o|)$, $2\theta_{max}$ = 56°], 607 parameters. CCDC 1498756.

The X-ray crystal structure of 5

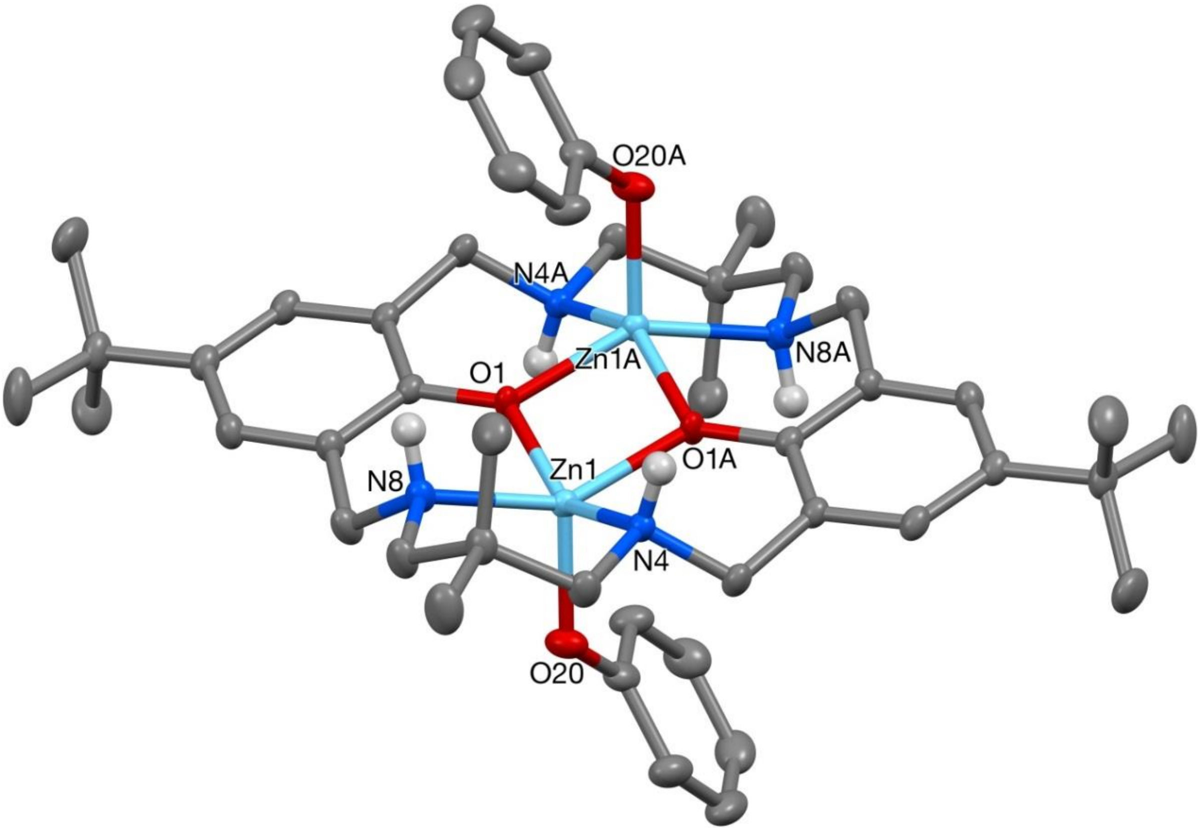

Figure S33. The structure of the C_i-symmetric complex present in the crystals of **5** (50% probability ellipsoids).

The complex in the structure of **5** was found to be situated across a centre of symmetry. The unique, C30-based, included dichloromethane solvent molecule was found to be disordered. Two orientations were identified of *ca*. 88 and 12% occupancy, their geometries were optimised, the thermal parameters of adjacent atoms were restrained to be similar, and only the non-hydrogen atoms of the major occupancy orientation were refined anisotropically (those of the minor occupancy orientation were refined isotropically). Only one of the two O40-based water protons could be reliably located from a Δ*F* map, and as a result the atom list for the asymmetric unit is low by 1H (and that for the unit cell low by 2H) compared to what is actually presumed to be present. The two unique N–H hydrogen atoms were located from Δ*F* maps and refined freely subject to an N–H distance constraint of 0.90 Å.

Crystal data for **5**: $C_{46}H_{64}N_4O_4Zn_2\cdot2(CH_2Cl_2)\cdot2(H_2O)$, M = 1073.64, triclinic, P-1 (no. 2), a = 9.3120(3), b = 10.9439(4), c = 13.4299(5) Å, α = 89.990(3), β = 71.865(3), γ = 82.514(3)°, V = 1288.38(8) Å^3, Z = 1 [C_i symmetry], D_c = 1.384 g cm^{-3}, μ(Mo-Kα) = 1.188 mm^{-1}, T = 173 K, colourless

blocks, Oxford Diffraction Xcalibur 3 diffractometer; 8396 independent measured reflections (R_{int} = 0.0186), F^2 refinement,[X1] R_1(obs) = 0.0311, wR_2(all) = 0.0791, 7271 independent observed absorption-corrected reflections [$|F_o| > 4\sigma(|F_o|)$, $2\theta_{max} = 65°$], 314 parameters. CCDC 1498757.

References

[1] A. Buchard, F. Jutz, M. R. Kember, A. J. P. White, H. S. Rzepa, C. K. Williams, *Macromolecules* **2012**, *45*, 6781-6795.

[2] (a) Y. J. Luo, W. Y. Li, D. Lin, Y. M. Yao, Y. Zhang, Q. Shen, *Organometallics* **2010**, *29*; (b) SHELX-97, G. M. Sheldrick, *Acta Cryst.* **2008**, *A64*, 112-122; (c)
